# Supplementary material for: Polyreactive Broadly Neutralizing B cells Are Selected to Provide Defense against Pandemic Threat Influenza Viruses
Source: Immunity. 2020 Dec 15;53(6):1230–1244.e5. doi: 10.1016/j.immuni.2020.10.005 (PMC7772752; doi:10.1016/j.immuni.2020.10.005)
Supplement: Document S1. Figures S1–S6 and Tables S1 and S2 [file mmc1.pdf]

Immunity, Volume 53

Supplemental Information

Polyreactive Broadly Neutralizing B cells Are Selected to Provide Defense against Pandemic Threat Influenza Viruses

Jenna J. Guthmiller, Linda Yu-Ling Lan, Monica L. Fernández-Quintero, Julianna Han, Henry A. Utset, Dalia J. Bitar, Natalie J. Hamel, Olivia Stovicek, Lei Li, Micah Tepora, Carole Henry, Karlynn E. Neu, Haley L. Dugan, Marta T. Borowska, Yao-Qing Chen, Sean T.H. Liu, Christopher T. Stamper, Nai-Ying Zheng, Min Huang, Anna-Karin E. Palm, Adolfo García-Sastre, Raffael Nachbagauer, Peter Palese, Lynda Coughlan, Florian Krammer, Andrew B. Ward, Klaus R. Liedl, and Patrick C. Wilson

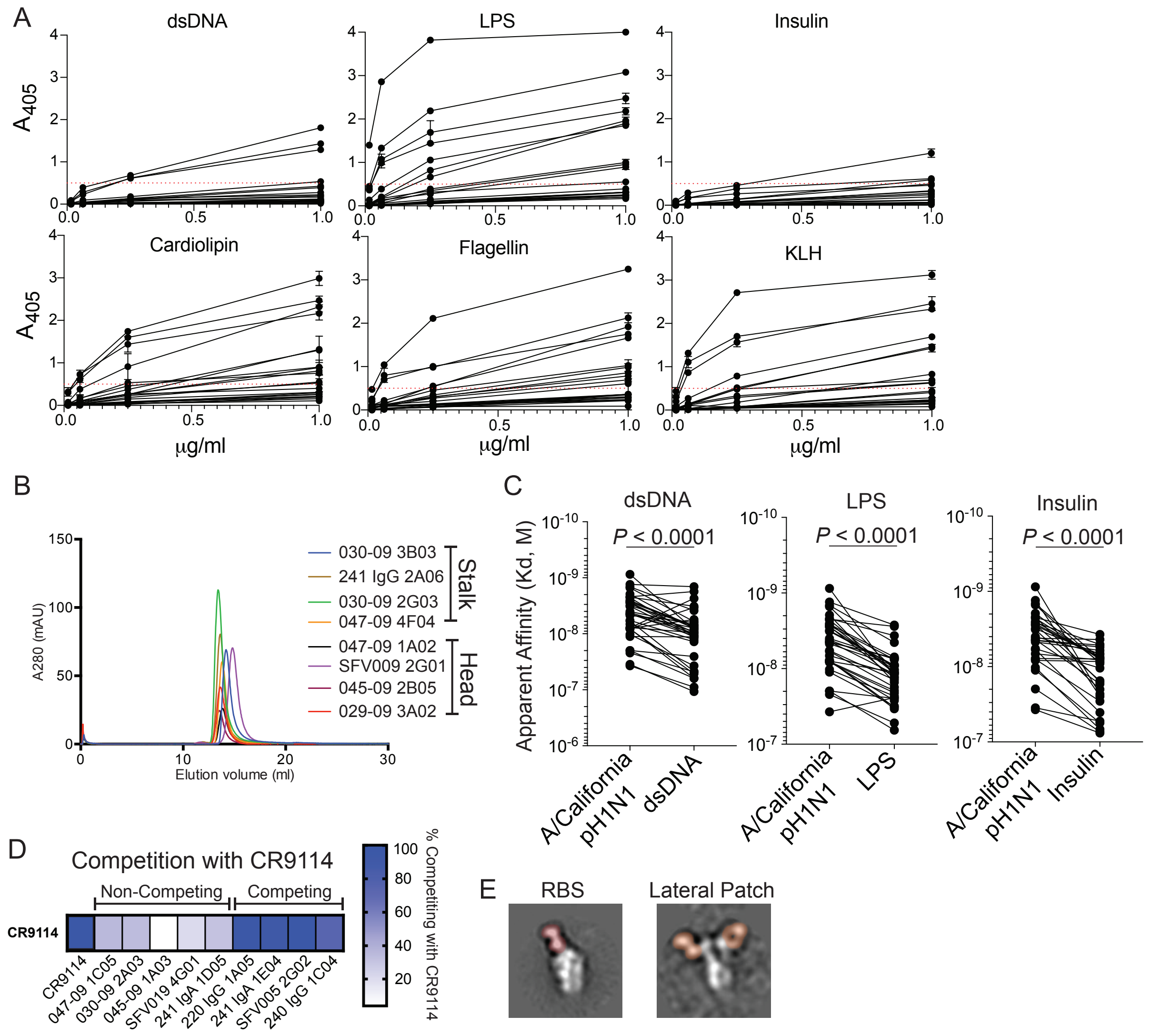

Figure S1: Polyreactive binding of influenza virus-binding antibodies. Related to Figure 1. (**A**) Polyreactivity ELISA example graphs. Representative of 22 antibodies tested for polyreactivity against the 6 antigens used in the polyreactivity ELISA panel. (**B**) Size exclusion chromatography of polyreactive mAbs indicating that polyreactive mAbs are monomers and do not form aggregates. (**C**) Paired apparent affinity (K_d) of polyreactive mAbs binding to A/California/7/2009 (pH1N1) virus and dsDNA (n=37), insulin (n=30), or LPS (n=36). Each line connects the same mAb. (**D**) Representative competition ELISA results of mAbs competing or not competing with CR9114, an antibody that specifically targets the BN stalk epitope. (**E**) Representative negative stain electron microscopy of an RBS binding antibody (SFV018 2D01 fab in red) and lateral patch binding antibody (045-09 2B05 fab in orange). Data in **C** were analyzed by paired non-parametric Wilcoxon matched-pairs signed rank Tests. Limit of Detection (L.O.D.) represented as dashed red line.

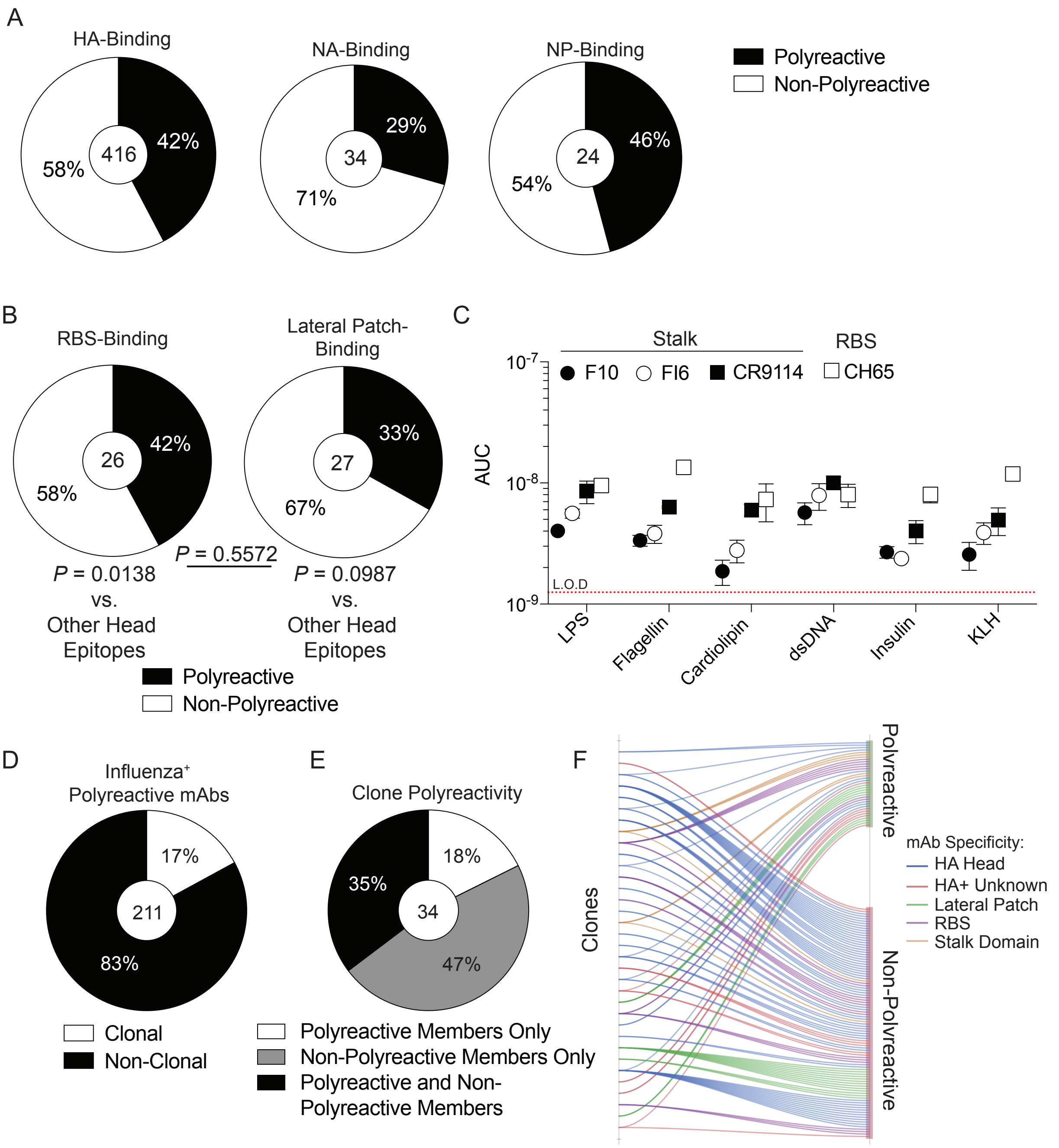

Figure S2: Polyreactivity of antigen-specific mAbs. Related to Figure 1. (**A**) Proportion of mAbs binding HA, NA, and NP that are polyreactive. (**B**) Proportion of RBS or lateral patch-binding mAbs that are polyreactive. (**C**) Polyreactivity of 4 published broadly neutralizing mAbs. The number in the center of pie graphs indicates the number of mAbs tested. Each antibody was tested in duplicate twice and the data are mean ± S.E.M. (**D**) Proportion of influenza virus positive polyreactive mAbs that are part of a clonal expansion. (**E**) Proportion of clones that only have polyreactive members, non-polyreactive members, or a mix of polyreactive and non-polyreactive

members. (**F**) Polyreactivity of clones based on antigen specificity. Each clone line on the left-hand side is one clonal expansion against HA (n=34 clones). For data in **A**, **B**, and **D**, the number in the center of each pie-graph is the number of mAbs tested. For data in **E**, the number in the center of the pie graph is the number of influenza virus specific clones analyzed. Data in **B** were analyzed by Fisher's Exact Test relative to other head epitope data in **Figure 1D**. Limit of Detection (L.O.D.) represented as dashed red line.

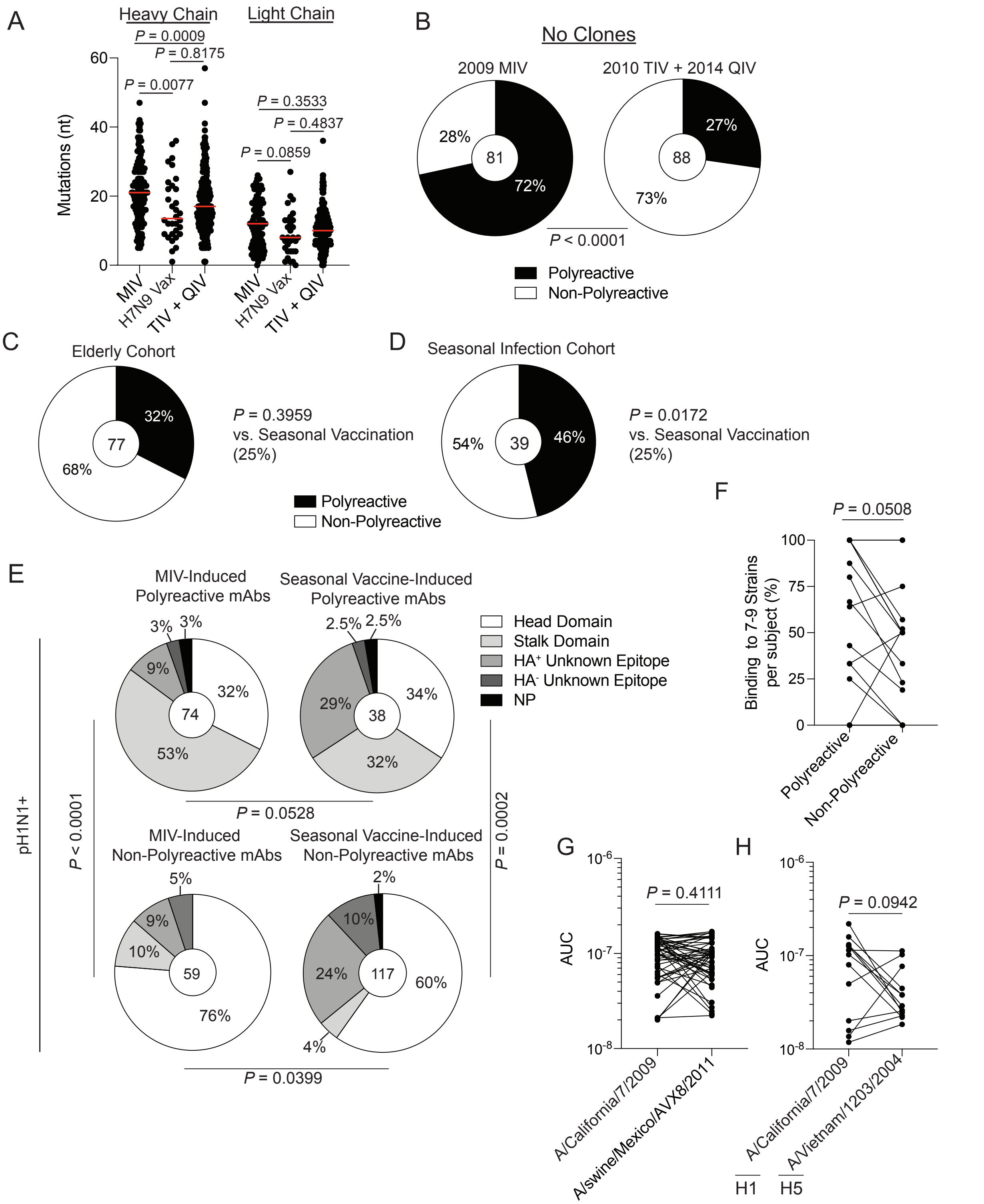

Figure S3: Polyreactive mAb induction by different influenza exposures and cross-reactivity of polyreactive mAbs. Related to Figure 2 and Figure 3. (**A**) Number of nucleotide mutations of heavy and light chains of mAbs generated from the 2009 MIV (heavy n=131; light n=123), H7N9 vaccine (heavy n=32; light n=31), and seasonal vaccination (TIV+QIV; heavy

n=259; light n=249). (**B**) Proportion of pH1N1$^+$ mAbs that are polyreactive from individuals vaccinated with the 2009 MIV or 2010-2011 TIV + 2014-2015 QIV, excluding any clonal expansions. (**C**-**D**) MAbs isolated from elderly subjects (≥ 65 years old) immunized with seasonal influenza vaccines (**C**) or from adults infected with seasonal influenza A viruses (**D**) were tested for polyreactivity. (**E**) Epitope targeting of polyreactive and non-polyreactive mAbs induced by the 2009 MIV (left) or seasonal vaccination (right). (**F**) Proportion of polyreactive and non-polyreactive mAbs per subject (n=12) binding to 7-9 H1N1 strains, based on data in **Figure 3A**. Each line connects the proportion of polyreactive and non-polyreactive mAbs binding 7-9 H1N1 strains from each subject. (**G**) Binding affinity (as shown as AUC) of polyreactive mAbs (n=50) induced by the 2009 MIV against A/California/7/2009 and A/swine/Mexico/AVX8/2011 (H1N2). (**H**) Binding affinity (as shown as AUC) polyreactive mAbs (n=13) induced by the 2009 MIV and the 2014 QIV against A/California/7/2009 and A/Vietnam/1203/2004 recombinant H5. For data in **A**, each symbol represents one mAb and the red bar is the median. Lines in **F** and **G** connect the same mAb binding A/California/7/2009 and A/swine/Mexico/AVX8/2011 (**F**) or A/Vietnam/1203/2004 rH5 (**G**). For data in **B**-**E**, the number in the center of each pie graph is the number of mAbs tested. Data in **A** were analyzed by a non-parametric Kruskal-Wallis Test, data in **B**-**D** were analyzed by Fisher's Exact Test, data in **E** were analyzed by using Chi-square Tests, and data in **F**-**H** were analyzed by a paired non-parametric Wilcoxon matched-pairs signed rank Test.

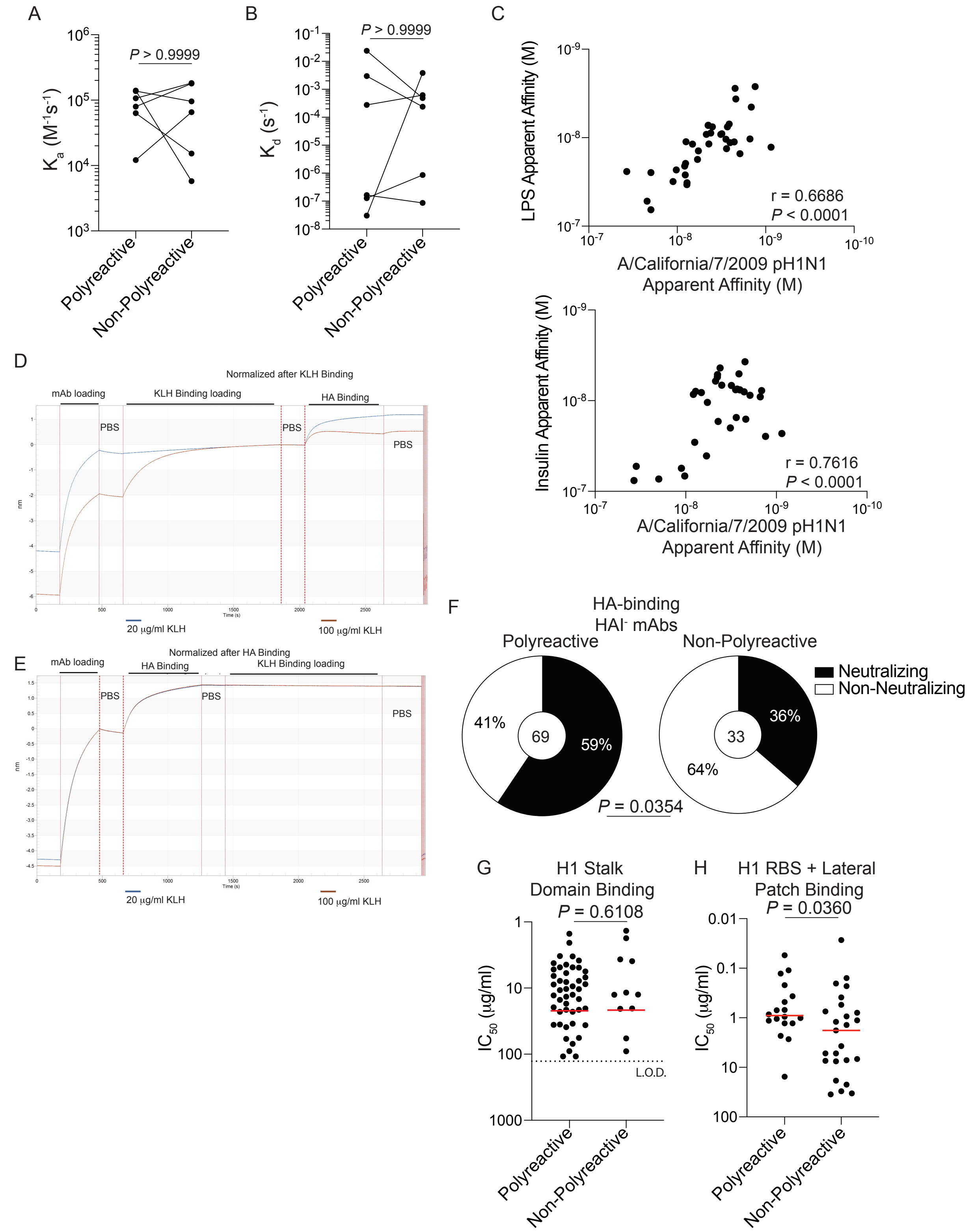

Figure S4: Polyreactivity augments viral binding and neutralization. Related to Figure 4. (**A-B**) K_a (**A**) and K_d (**B**) of polyreactive and non-polyreactive mAbs from the same clone binding to A/California/7/2009 HA. Each line connects polyreactive and non-polyreactive clonal members

(n=6). (**C**) Spearman Correlation of the apparent affinity (K_d) of polyreactive mAb binding to A/California/7/2009 virus and LPS (top; n=36) or Insulin (bottom; n=30). (**D**-**E**) Using biolayer interferometry, a Protein A sensor was loaded with SFV005 2G02 (polyreactive mAb). (**D**) The sensor was then dipped in 20 μg/ml or 100 μg/ml of KLH, followed by 10 μg/ml A/California/7/2009 HA. (**E**) After SFV005 2G02, the sensor was dipped into 10 μg/ml A/California/7/2009 HA, and then dipped in 20 μg/ml or 100 μg/ml of KLH. Data are representative of 10 mAbs. The assays were performed twice for each antibody. (**F**) Polyreactive and non-polyreactive antibodies targeting HA^+ HAI^- epitopes were tested for neutralization against A/California/7/2009. Proportion of polyreactive and non-polyreactive antibodies that are neutralizing. (**G**-**H**) Neutralization potency (IC_{50}) against A/California/7/2009 virus of polyreactive (n=47) and non-polyreactive (n=11) mAbs targeting the stalk domain (**G**) and of polyreactive (n=18) and non-polyreactive (n=25) mAbs targeting the RBS and lateral patch (**H**). For data in **C**, **G**, and **H**, each symbol represents one mAb and the red bar indicates the median. For data in **F**, the number in the center of each pie graph is the number of mAbs tested. Data in **A** and **B** were analyzed by a paired non-parametric Wilcoxon matched-pairs signed rank Test. Data in **F** were analyzed using a Fisher's Exact Test and data in **G** and **H** were analyzed using a non-parametric Mann-Whitney Test. Limit of Detection (L.O.D.) represented as dashed black line.

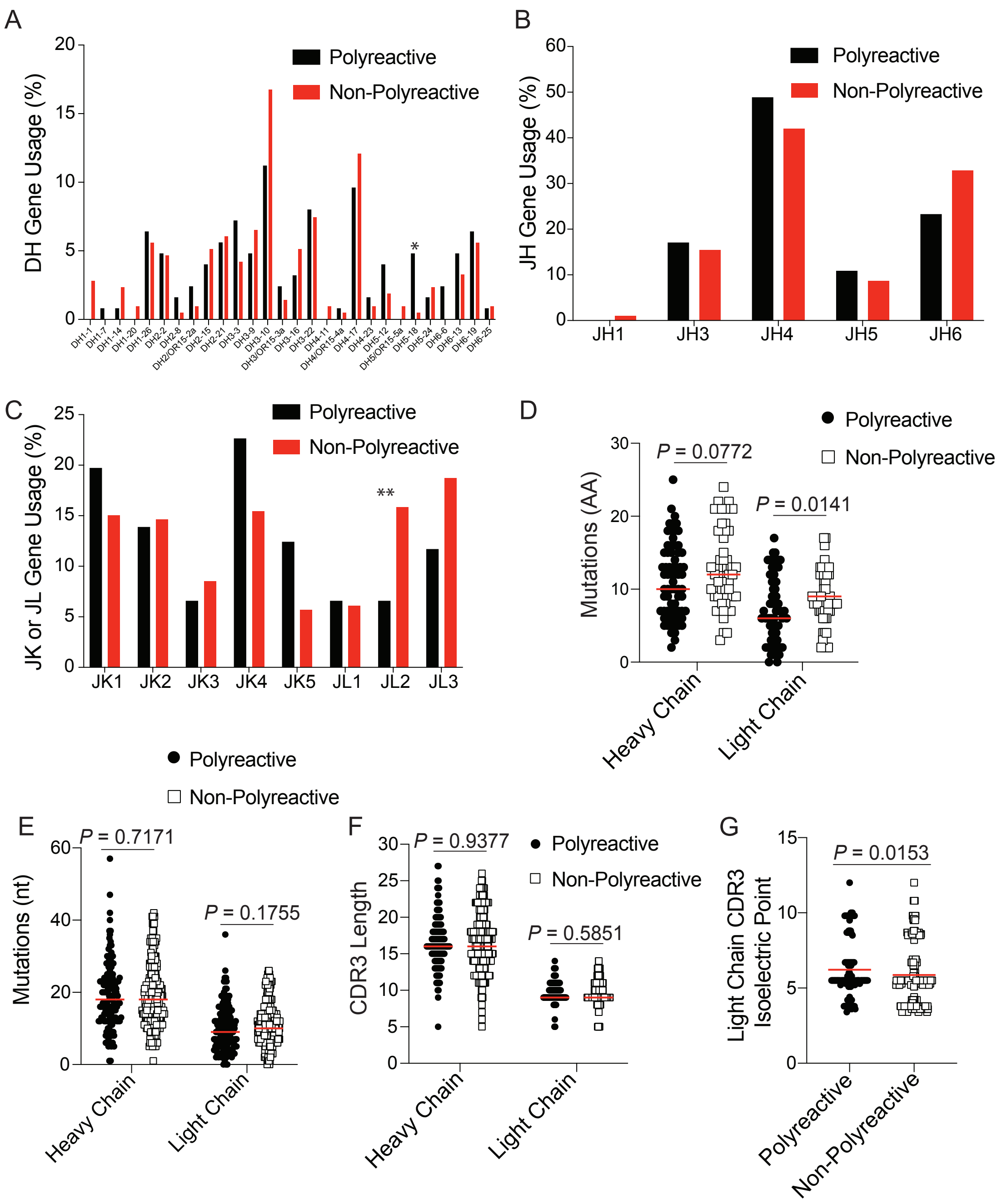

Figure S5: Repertoire and biochemical characteristics of polyreactive and non-polyreactive antibodies. Related to Figure 5. (**A-B**) DH (**A**) and JH (**B**) gene usage by polyreactive and non-polyreactive antibodies. (**C**) JK or JL gene usage by polyreactive and non-polyreactive antibodies. (**D**) Somatic hyper mutations (amino acid changes) of polyreactive (heavy n=71; light 68) and non-polyreactive (heavy n=55; light n=53) mAbs induced by the 2009 MIV. (**E**) Somatic hypermutations (nucleotide mutations) of heavy and light chains of all polyreactive (n=137) and non-polyreactive (n=246) mAbs. (**F**) Heavy chain and light chain CDR3 length of

polyreactive (n=137) and non-polyreactive mAbs (heavy n=245; light n=246). (**G**) Light chain CDR3 isoelectric point of polyreactive (n=137) and non-polyreactive (n=246) mAbs. For data in **D**-**G**, each symbol represents one mAb and the red bar indicates the median. Data in **A**-**C** were analyzed by Fisher's Exact Tests, and data in **D**-**G** were analyzed by unpaired non-parametric Mann-Whitney Tests. Each symbol represents a single antibody. * $P \leq 0.05$; ** $P \leq 0.01$

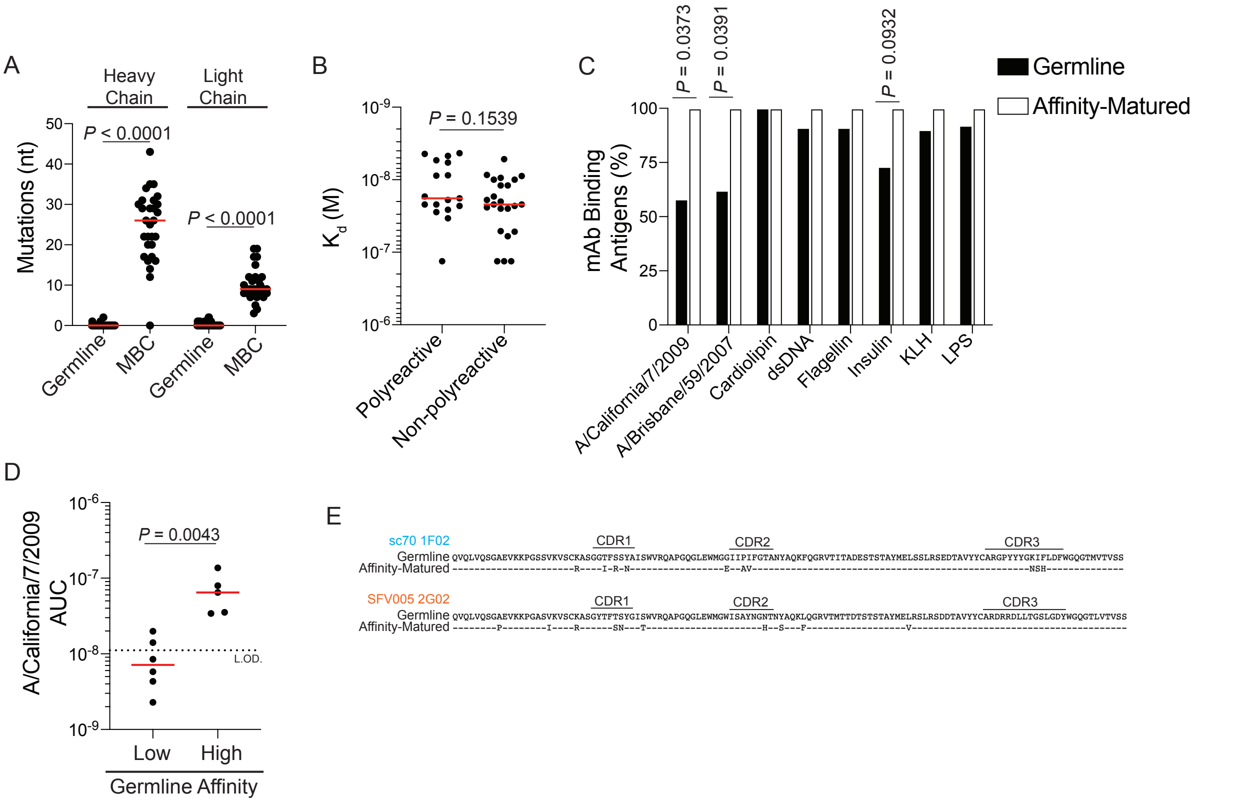

Figure S6: Germline precursors of broadly-reactive antibodies are polyreactive. Related to Figure 6. (**A**) Somatic hypermutations (nucleotide mutations) of heavy and light chains of stalk domain-binding germline (n=50) and MBC (n=29) mAbs tested in **Figure 6A**. (**B**) Affinity of polyreactive (n=17) and non-polyreactive (n=23) germline mAbs binding the stalk domain. (**C**) Proportion of reverted germline mAbs generated from affinity-matured polyreactive and corresponding affinity-matured mAbs binding influenza viruses and polyreactive panel antigens. (**D**) Area under the curve (AUC) of reverted germline mAbs categorized as high (n=5) or low (n=6) affinity binding to A/California/7/2009 related to **Figure 6E**. (**E**) Heavy chain sequences of germline and affinity-matured sc70 1F02 and SFV005 2G02. For data in **A**, **B**, and **D**, each symbol represents one mAb and the red bar indicates the median. Data in **A**, **B**, and **D** were analyzed by an unpaired non-parametric Mann-Whitney Test and data in **C** were analyzed by Fisher's Exact Test.

Table S1: Influenza vaccination and infection and influenza-negative naïve B cell and MBC cohorts. Related to STAR Methods.

Cohort	# of Subjects	# of mAbs	Average # mAbs per subject (range)	Reference
pH1N1 MIV	11	133	12 (1 – 29)	(Andrews et al., 2015a)
2010-2011 TIV	12	48	4 (1 – 6)	(Andrews et al., 2015a)
2014-2015 QIV	8	166	21 (8 – 53)	(Neu et al., 2019)
H7N9 LAIV/IIV	5	31	6 (2 – 16)	(Henry Dunand et al., 2016)
Elderly pre-H1N1 TIV	13	77	6 (1 – 21)	(Henry et al., 2019)
Chimeric HA Vaccine Germline	12	50	4 (1 – 16)	(Bernstein et al., 2019)
Chimeric HA Vaccine MBCs	12	29	2 (1 – 11)	(Bernstein et al., 2019)
2014-2015 H3N2 Infected	3	18	6 (2 – 10)	(Chen et al., 2018)
2015-2016 H1N1 Infected	4	21	5 (1 – 10)	(Chen et al., 2018)
Influenza-Negative Naïve B cells	3	52	17 (11 – 28)	(Duty et al., 2009)
Influenza-Negative MBCs	4	56	14 (7 – 22)	(Koelsch et al., 2007)

Table S2: Subject demographics for cohorts. Related to STAR Methods. *No demographic information was obtained from the H7N9 LAIV/IIV cohort and the influenza-negative naïve B cell and MBC cohorts.

Cohort	# of Subjects	Male (%)	Mean Age [Range]
pH1N1 MIV	11	36.4	41.1 (24 – 64)
2010-2011 TIV	12	58.3	29.3 (23 – 43)
2014-2015 QIV	8	37.5	29.9 (24 – 34)
Elderly pre-H1N1 TIV	13	53.8	75.7 (71 – 89)
Chimeric HA Vaccine Germline	12	25	27.7 (20 – 37)
Chimeric HA Vaccine MBCs	12	25	30.9 (24 – 36)
2014-2015 H3N2 Infected	3	66.7	43 (34 – 49)
2015-2016 H1N1 Infected	4	31.25	31.3 (23 – 46)
