# Supplementary material for: Polyreactive Broadly Neutralizing B cells Are Selected to Provide Defense against Pandemic Threat Influenza Viruses
Source: Immunity. 2020 Dec 15;53(6):1230–1244.e5. doi: 10.1016/j.immuni.2020.10.005 (PMC7772752; doi:10.1016/j.immuni.2020.10.005)
Supplement: Document S2. Article plus Supplemental Information [file mmc2.pdf]

Article

Immunity

Polyreactive Broadly Neutralizing B cells Are Selected to Provide Defense against Pandemic Threat Influenza Viruses

Graphical Abstract

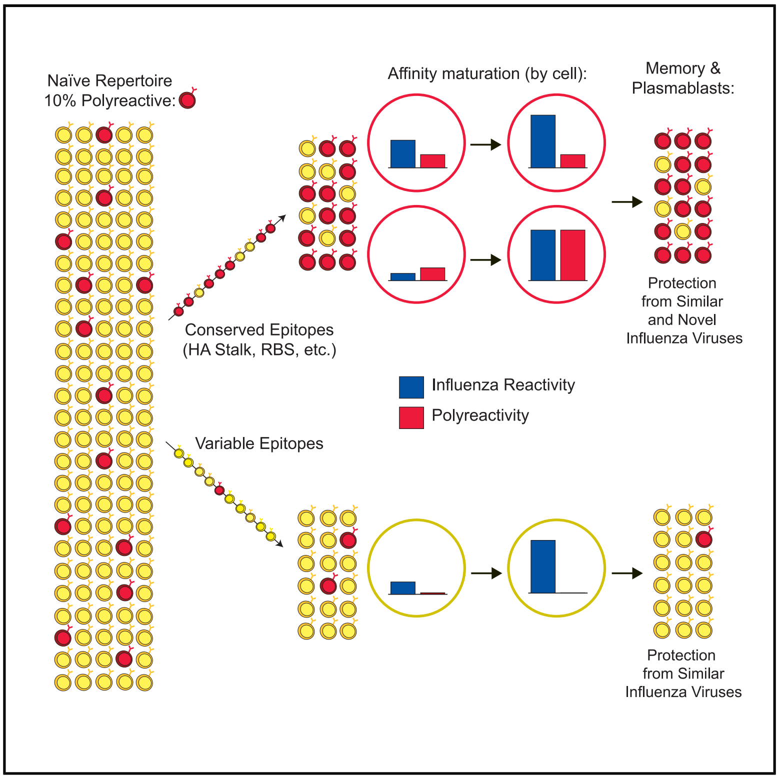

Authors

Jenna J. Guthmiller, Linda Yu-Ling Lan, Monica L. Fernández-Quintero, ..., Andrew B. Ward, Klaus R. Liedl, Patrick C. Wilson

Correspondence

wilsonp@uchicago.edu

In Brief

Polyreactivity is a common feature of antibodies, but little is known about the selection, function, and role of polyreactive B cells against pathogens. Guthmiller et al. demonstrate polyreactive B cells are selected against broadly neutralizing epitopes of influenza viruses and are linked to increased viral-binding breadth, affinity, and antibody flexibility.

Highlights

- Antibodies targeting conserved hemagglutinin epitopes are polyreactive
- Polyreactive antibodies are preferentially induced by novel influenza viruses
- Polyreactivity increases antibody flexibility, affinity, and neutralization potency
- Polyreactive naive B cells are selected into the memory B cell pool

Guthmiller et al., 2020, Immunity *53*, 1230–1244
December 15, 2020 © 2020 The Authors. Published by Elsevier Inc.
https://doi.org/10.1016/j.immuni.2020.10.005

CellPress
OPEN ACCESS

Immunity

Article

Polyreactive Broadly Neutralizing B cells Are Selected to Provide Defense against Pandemic Threat Influenza Viruses

Jenna J. Guthmiller,[1] Linda Yu-Ling Lan,[2] Monica L. Fernández-Quintero,[3] Julianna Han,[4] Henry A. Utset,[1] Dalia J. Bitar,[1] Natalie J. Hamel,[1] Olivia Stovicek,[1] Lei Li,[1] Micah Tepora,[1] Carole Henry,[1] Karlynn E. Neu,[1,2] Haley L. Dugan,[2] Marta T. Borowska,[5] Yao-Qing Chen,[1] Sean T.H. Liu,[6] Christopher T. Stamper,[2] Nai-Ying Zheng,[1] Min Huang,[1] Anna-Karin E. Palm,[1] Adolfo García-Sastre,[6,7,8,9] Raffael Nachbagauer,[6] Peter Palese,[6] Lynda Coughlan,[6,10] Florian Krammer,[6] Andrew B. Ward,[4] Klaus R. Liedl,[3] and Patrick C. Wilson[1,2,11,*]

[1]Department of Medicine, Section of Rheumatology, University of Chicago, Chicago, IL 60637, USA
[2]Committee on Immunology, University of Chicago, Chicago, IL 60637, USA
[3]Center for Molecular Biosciences Innsbruck, Institute of General, Inorganic and Theoretical Chemistry, University of Innsbruck, 6020 Innsbruck, Austria
[4]Department of Integrative Structural and Computational Biology, The Scripps Research Institute, La Jolla, CA 92037, USA
[5]Department of Biochemistry and Molecular Biology, University of Chicago, Chicago, IL 60637, USA
[6]Department of Microbiology, Icahn School of Medicine at Mount Sinai, New York, NY 10029, USA
[7]Department of Medicine, Division of Infectious Diseases, Icahn School of Medicine at Mount Sinai, New York, NY 10029, USA
[8]Global Health and Emerging Pathogens Institute, Icahn School of Medicine at Mount Sinai, New York, NY 10029, USA
[9]The Tisch Cancer Center, Icahn School of Medicine at Mount Sinai, New York, NY 10029, USA
[10]Department of Microbiology and Immunology and Center for Vaccine Development and Global Health, University of Maryland School of Medicine, Baltimore, MD 21201, USA
[11]Lead Contact
*Correspondence: wilsonp@uchicago.edu
https://doi.org/10.1016/j.immuni.2020.10.005

SUMMARY

Polyreactivity is the ability of a single antibody to bind to multiple molecularly distinct antigens and is a common feature of antibodies induced upon pathogen exposure. However, little is known about the role of polyreactivity during anti-influenza virus antibody responses. By analyzing more than 500 monoclonal antibodies (mAbs) derived from B cells induced by numerous influenza virus vaccines and infections, we found mAbs targeting conserved neutralizing influenza virus hemagglutinin epitopes were polyreactive. Polyreactive mAbs were preferentially induced by novel viral exposures due to their broad viral binding breadth. Polyreactivity augmented mAb viral binding strength by increasing antibody flexibility, allowing for adaption to imperfectly conserved epitopes. Lastly, we found affinity-matured polyreactive B cells were typically derived from germline polyreactive B cells that were preferentially selected to participate in B cell responses over time. Together, our data reveal that polyreactivity is a beneficial feature of antibodies targeting conserved epitopes.

INTRODUCTION

Broadly neutralizing antibodies targeting influenza viruses largely bind conserved epitopes found within the receptor-binding site (RBS), lateral patch, and stalk domain of the viral surface protein hemagglutinin (HA) (Dreyfus et al., 2012; Whittle et al., 2011; Ekiert et al., 2012; Ekiert et al., 2009; Raymond et al., 2018). The induction of broadly neutralizing antibodies is the goal of a universal vaccine that can protect against >75% of influenza A viruses (Paules et al., 2017). No universal influenza virus vaccine yet exists, and annual vaccinations against circulating strains are still recommended. Current seasonal influenza virus vaccines largely stimulate strain-specific antibodies targeting the polymorphic head domain of HA and provide narrow protection against circulating strains (Andrews et al., 2015a). In contrast, exposure to the antigenically distinct 2009 pandemic H1N1 (pH1N1) and novel avian influenza viruses, including H5N1 and H7N9, induced antibodies targeting conserved epitopes found within the HA stalk domain (Andrews et al., 2015a; Li et al., 2012; Henry Dunand et al., 2016; Ellebedy et al., 2014; Wrammert et al., 2011), which are correlated with protection against influenza virus infection in humans (Ng et al., 2019).

Broadly neutralizing antibodies against influenza viruses are rarely induced by seasonal vaccination for unclear reasons. The HA stalk domain is immuno-subdominant relative to the

 © 2020 The Authors. Published by Elsevier Inc.
This is an open access article under the CC BY license (http://creativecommons.org/licenses/by/4.0/).

CellPress
OPEN ACCESS

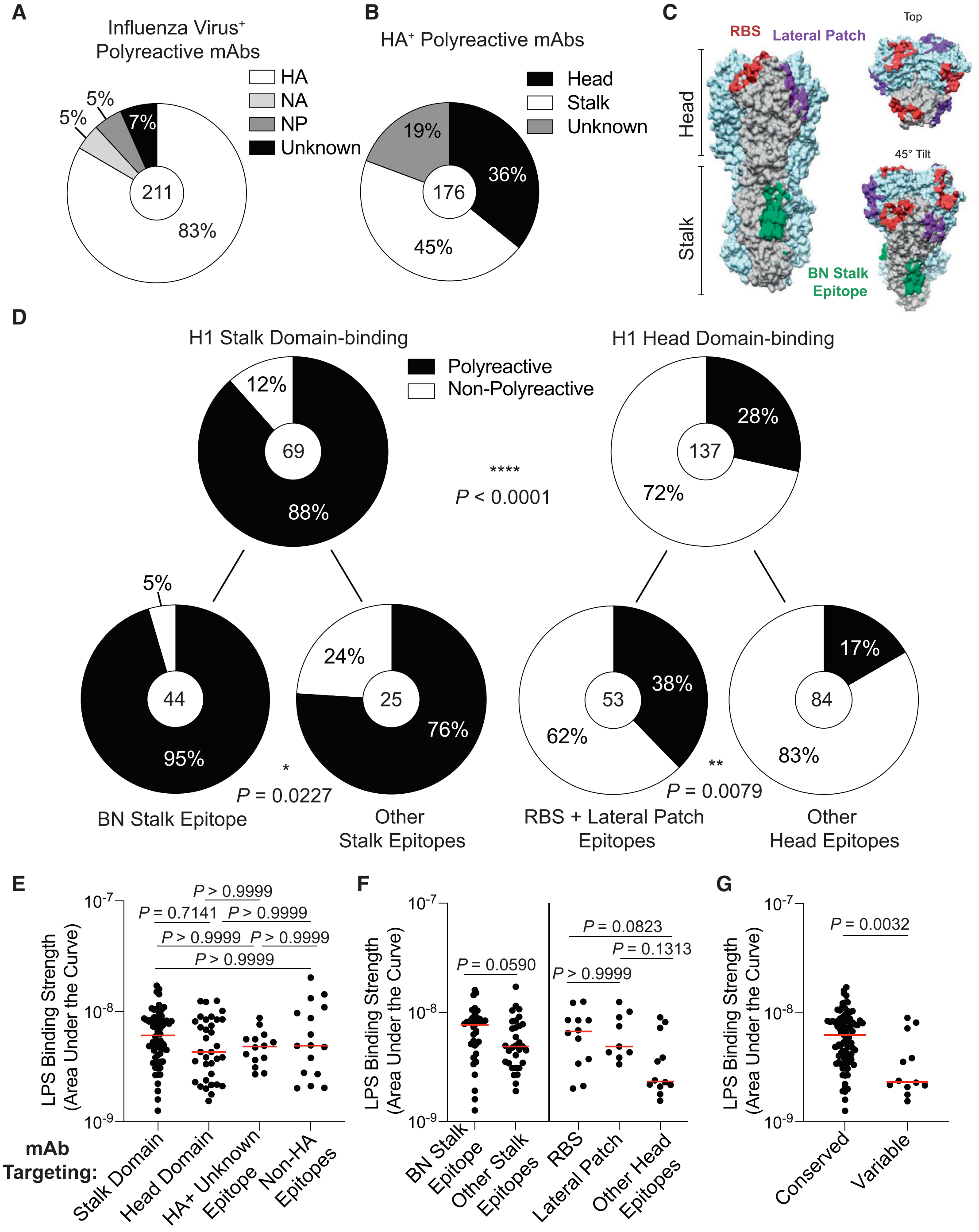

Figure 1. Polyreactive mAbs Target Conserved Epitopes on HA

(A and B) Proportion of polyreactive mAbs binding distinct influenza antigens (A) and HA domains (B).

(C) Structure of A/California/7/2009 HA (PDB: 4M4Y) showing the footprint of three mAbs binding conserved epitopes: CH65 (RBS; PDB: 5UGY), Fab6649 (lateral patch; PDB: 5W6G), and CR9114 (BN stalk epitope; PDB: 4FQI).

(D) Proportion of stalk domain mAbs and head domain mAbs that are polyreactive, further broken down by specific epitopes targeted (bottom).

(E–G) Polyreactive mAb binding strength to LPS (AUC) of mAbs targeting distinct antigens and antigen domains (stalk domain n = 66; head domain n = 34; HA+ unknown epitope n = 14; non-HA epitopes n = 16) (E), distinct epitopes of HA stalk domain (BN stalk epitope n = 35; other stalk epitopes n = 31) and head domain (RBS n = 12; lateral patch n = 9; other head epitopes n = 12) (F), and conserved epitopes of HA (stalk domain, RBS, and lateral patch; n = 87) and variable epitopes of the HA head (n = 12) (G).

(legend continued on next page)

HA head, potentially due to poor immunogenicity and steric hindrance (Tan et al., 2019; Angeletti et al., 2019; Andrews et al., 2015a). The RBS epitope is also immuno-subdominant, likely because the conserved residues of the RBS pocket are buried (Schmidt et al., 2015; Ekiert et al., 2012). Additionally, pre-existing titers of antibodies to variable epitopes on drifted influenza virus strains could mask conserved HA epitopes, sterically hindering the activation of memory B cells (MBCs) to these conserved epitopes (Zarnitsyna et al., 2015; Andrews et al., 2015b). Understanding how to continually induce broadly neutralizing antibodies against conserved HA epitopes will enhance the development of a universal influenza vaccine.

Broadly neutralizing antibodies against HIV and influenza viruses are commonly polyreactive, defined herein as the ability of a single antibody to bind multiple molecularly distinct antigens, including self-antigens. Nearly 70% of broadly neutralizing HIV-binding antibodies are polyreactive (Dennison et al., 2011; Haynes et al., 2005). Similarly, antibodies binding the HA stalk domain are commonly polyreactive (Andrews et al., 2015a; Bajic et al., 2019). Due to the inherent self-reactivity of polyreactive antibodies, B cells expressing polyreactive antibodies could be counter-selected during development or become anergic in the periphery. Therefore, polyreactivity could limit the induction of broadly neutralizing antibody responses against conserved epitopes such as the HA stalk domain.

Polyreactivity is a common feature of all B cells, with polyreactive B cells comprising 6% of the naive B cell repertoire and ~25% of the IgG^+ MBC pool (Tiller et al., 2007). To overcome extreme antigenic variation, B cell receptors (BCRs) might have evolved to harbor polyreactivity to increase recognition and neutralization of divergent influenza viruses as a stopgap measure rather than relying on slower and lower-affinity *de novo* B cell responses. However, the precise role of polyreactivity in antigen-specific B cell responses remains unclear. Additionally, polyreactive antibodies induced by HIV infection and influenza virus vaccination are highly mutated (Liu et al., 2015; Andrews et al., 2015a; Scheid et al., 2009), indicating polyreactive B cells against HIV and influenza viruses are predominately selected within the germinal center and are not just the product of innate-like B cells.

Here, we report that polyreactivity is a common feature of broadly neutralizing monoclonal antibodies (mAbs) targeting conserved influenza virus epitopes of both the HA stalk domain and the conserved RBS and lateral patch of the head domain. Influenza virus-binding polyreactive mAbs are highly cross-reactive among influenza viruses and are the major component of the antibody response induced by novel influenza virus exposures, indicating that polyreactive antibody responses are a critical first line of defense against novel influenza virus strains. Polyreactivity augments viral binding strength by increasing antibody flexibility within the variable regions, in contrast to the traditional "lock-in-key" mechanism of antibody paratope binding to a single antigen. We also report that polyreactivity is inherent to naive B cells targeting the stalk domain, and polyreactive B cells are selected into the broadly neutralizing MBC pool. Together, these findings reveal polyreactivity to be a hallmark of broadly neutralizing B cells induced by influenza viruses.

RESULTS

MAbs Targeting Broadly Neutralizing Epitopes of HA Are Polyreactive

Little is known about the influenza reactivity of polyreactive mAbs induced by influenza virus exposure. To address this question, we collected peripheral blood mononuclear cells (PBMCs) from humans vaccinated with influenza virus vaccines or that were naturally infected with influenza viruses and cloned mAbs from sorted transient antibody-secreting plasmablasts or HA-baited MBCs. To test for polyreactivity, we performed a commonly used ELISA assay (Wardemann et al., 2003; Bunker et al., 2017) for mAb binding to six molecularly distinct antigens: cardiolipin, double-stranded DNA (dsDNA), *Salmonella enterica* flagellin, human insulin, keyhole limpet hemocyanin (KLH), and *Escherichia coli* lipopolysaccharide (LPS) (Figure S1A). Polyreactive mAb binding to antigens in our assay could not be explained by non-specific binding of antibody aggregates, because polyreactive mAbs were monomeric by size-exclusion chromatography (Figure S1B). Notably, polyreactive mAbs induced by influenza virus exposure had stronger apparent affinity for influenza viruses than did non-specific antigens used in the polyreactivity panel (Figure S1C), suggesting that mAbs were derived from B cells that were specifically activated by influenza virus vaccination or infection. The influenza virus epitopes being targeted by mAbs were determined by antigen-specific ELISAs, competition ELISAs, or electron microscopy (Figures S1D and S1E).

The vast majority (83%) of polyreactive mAbs targeted the major surface glycoprotein HA, but 17% recognized subdominant antigens such as nucleoprotein (NP) and neuraminidase (NA) (Figure 1A). Conversely, the majority of mAbs targeting HA, NA, and NP were not polyreactive (Figure S2A), suggesting that polyreactivity is not a dominant feature of influenza virus-reactive mAbs. A breakdown of polyreactive HA-binding mAbs revealed that a plurality recognized the HA stalk domain, although a substantial 36% recognized epitopes on the HA head (Figure 1B). Of the total stalk and head domain-binding mAbs, 88% of stalk domain-binding and 28% of head domain-binding mAbs were polyreactive (Figures 1C and 1D; $p < 0.0001$), suggesting that polyreactivity is a dominant feature of stalk domain-binding mAbs, but not of head-binding mAbs. A breakdown of H1 stalk domain-binding mAbs revealed that 95% of mAbs recognizing the broadly neutralizing (BN) stalk epitope were polyreactive (Figure 1D). Similarly, 76% of mAbs targeting epitopes on the stalk domain other than the BN stalk epitope were polyreactive (Figure 1D; $p = 0.0227$). A breakdown of head-binding mAbs revealed that 38% of RBS- and lateral-patch-binding mAbs were polyreactive, whereas only 17% of

For data in (A), (B), and (D), the number in the center of each pie graph is the number of mAbs tested. For panels (E)–(G), each symbol represents one mAb and the red bar represents the median. Data for (D) were analyzed by Fisher's exact tests, data for (E) were analyzed by non-parametric Kruskal-Wallis test, and data for (F) and (G) were analyzed by non-parametric Mann-Whitney test. Statistical analysis for (G) was further tested by a Bootstrap analysis to validate biological significance. See also Figures S1 and S2.

CellPress
OPEN ACCESS

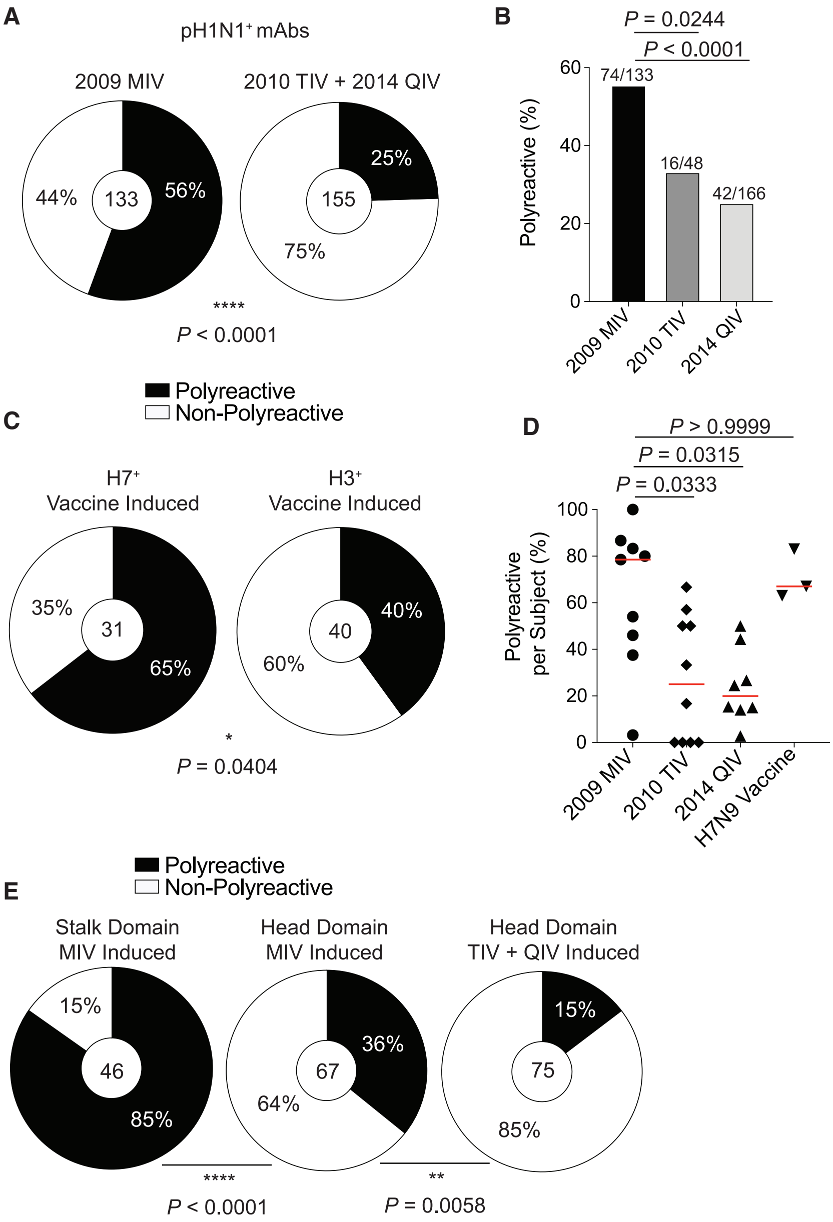

Figure 2. Novel Virus Exposures Induce Polyreactive mAbs

(A and B) Proportion of pH1N1⁺ mAbs (A) or all influenza⁺ mAbs (B) that are polyreactive from individuals vaccinated with the 2009 MIV or 2010 TIV + 2014 QIV.

(C) Proportion of H7⁺ or H3⁺ mAbs that are polyreactive from individuals vaccinated with an H7N9 LAIV/IIV or seasonal influenza vaccine (2010 TIV and 2014 QIV).

(D) Proportion of polyreactive influenza⁺ mAbs per subject by cohort (2009 MIV n = 9; 2010 TIV n = 10; 2014 QIV n = 8; H7N9 vaccine n = 3). Each symbol represents one subject, and the red bar represents the median. Only subjects with three or more mAbs were included in the analysis.

(E) Proportion of MIV stalk domain-induced, MIV head domain-induced, or seasonal vaccine head domain-induced mAbs that are polyreactive.

For data in (A), (C), and (E), the number in the center of each pie graph is the number of mAbs tested. For (B), the number on top of individual bars is the number of polyreactive mAbs out of total mAbs tested. Data for (A)–(C) and (E) were analyzed by Fisher's exact tests, and data for (D) were analyzed by a non-parametric Kruskal-Wallis test. See also Figure S3.

mAbs directed against other HA epitopes were polyreactive (Figure 1D; Figure S2B; p = 0.0079). Additionally, published broadly neutralizing mAbs (Corti et al., 2011; Sui et al., 2009; Dreyfus et al., 2012; Whittle et al., 2011) against the stalk domain (F10, FI6, CR9114) and the RBS (CH65) were also polyreactive (Figure S2C), suggesting that polyreactivity is a common feature of mAbs targeting conserved epitopes. Notably, 17% of polyreactive mAbs were part of a clonal lineage, and 35% of clones had members that were both polyreactive and non-polyreactive (Figures S2D and S2E). Whether or not particular clones were polyreactive was independent of epitope specificity (Figure S2F), indicating that clonal expansions did not bias our findings. To evaluate the degree of polyreactivity of mAbs targeting distinct HA epitopes, we next determined mAb apparent affinity against LPS. Polyreactive mAbs had similar apparent affinity for LPS regardless of influenza epitope binding (Figures 1E and 1F). When pooled together, polyreactive mAbs targeting conserved epitopes of HA had stronger apparent affinity for LPS relative to mAbs targeting variable HA epitopes (Figure 1G; p = 0.0032), indicating that mAbs against conserved epitopes were qualitatively more polyreactive. Together, these data uncover polyreactivity as a prevalent feature of mAbs against conserved HA epitopes.

Novel Influenza Virus Exposure Drives Polyreactive Antibody Responses

To determine whether exposure to a novel influenza virus drives polyreactive antibody responses, we tested polyreactivity of mAbs generated from subjects vaccinated with the 2009 pH1N1 (A/California/4/2009) monovalent influenza vaccine (MIV), with the MIV being the first exposure for these individuals to pH1N1, compared to mAbs cloned from a seasonal vaccine cohort in 2010 trivalent influenza vaccine (TIV) (Andrews et al., 2015a) and 2014 quadrivalent influenza vaccine (QIV) (Neu et al., 2019). Notably, we assayed mAbs generated from acutely activated plasmablasts that derive from recalled highly mutated MBCs (Figure S3A) and represented cross-reactive pre-existing immunity. 56% of pH1N1⁺ mAbs generated from 2009 MIV recipients were polyreactive, in comparison to only 25% of pH1N1⁺ mAbs generated from recipients of the 2010 TIV and 2014 QIV (Figures 2A and 2B; p < 0.0001). When excluding clonal expansions, 72% of pH1N1⁺ mAbs generated from 2009 MIV recipients were polyreactive, in comparison with only 27% of pH1N1⁺ mAbs induced by seasonal vaccination (Figure S3B). The proportion of polyreactive mAbs induced by the 2010 TIV

and 2014 QIV was similar to the proportion (23%) of polyreactive mAbs induced by pre-pH1N1 TIVs in healthy adults (Kaur et al., 2015), likely because the specificities of mAbs induced by pre-pH1N1 vaccination targeted similar polymorphic head epitopes as did antibodies induced by post-pH1N1 seasonal vaccines (Andrews et al., 2015a). We additionally tested whether polyreactive mAbs were induced by H7N9 vaccination, which recalled cross-reactive MBCs against conserved epitopes of the HA stalk domain (Henry Dunand et al., 2016). H7N9 vaccination primarily induced polyreactive mAbs, with nearly two-thirds of $H7^+$ mAbs being polyreactive, in contrast to two-fifths of H3N2-binding mAbs induced by seasonal vaccination (Figure 2C; $p = 0.0404$). We next tested whether mAbs isolated from elderly individuals after seasonal influenza vaccination or adults infected with seasonal influenza viruses were polyreactive, because mAbs induced under these circumstances tend to target conserved epitopes (Henry et al., 2019; Chen et al., 2018). mAbs isolated from elderly individuals did not have a propensity to be polyreactive (Figure S3C). Infected subjects had a higher proportion of polyreactive mAbs than did subjects that received a seasonal vaccine (Figure S3D), suggesting that the different antigenic targets and the inflammatory milieu of influenza virus infection promoted polyreactive B cell activation.

Our data demonstrated novel virus exposure preferentially induces polyreactive mAbs at an individual level (Figure 2D; $p = 0.0333$ MIV versus TIV; $p = 0.0315$ MIV versus QIV), likely as a consequence of antibodies being induced against conserved epitopes. Moreover, a significant proportion of polyreactive mAbs induced by seasonal and novel influenza vaccination targeted the stalk domain (Figure S3E), although stalk domain-binding mAbs are rarely induced by seasonal vaccination. In contrast, non-polyreactive mAbs induced by both the 2009 MIV and seasonal vaccination largely target the head domain (Figure S3E), confirming the non-polyreactive nature of most head-binding mAbs. Additionally, novel virus exposure induced more than a two-fold increase in polyreactive mAbs against the head domain relative to those induced by seasonal vaccination (Figure 2E; $p = 0.0058$), suggesting novel H1N1 exposure is inducing antibodies against conserved epitopes of the HA head. Together, these data indicate novel virus exposure induces polyreactive mAbs against conserved epitopes.

Polyreactive mAbs Are Broadly Cross-Reactive among Influenza Viruses

As the majority of mAbs against conserved HA epitopes were polyreactive, we next measured the viral binding breadth of polyreactive and non-polyreactive mAbs. Of the $pH1N1^+$ mAbs, 69% of polyreactive mAbs bound to nearly all H1N1 viruses tested, whereas non-polyreactive mAbs tended to be more pH1N1 strain specific (Figure 3A; $p < 0.0001$). Furthermore, 58% of polyreactive head domain-binding mAbs bound to nearly all H1N1 strains tested in comparison with only 38% of non-polyreactive mAbs, which displayed a plurality of pH1N1 strain-specific binding (Figure 3B; $p = 0.0035$). Additionally, polyreactive mAbs demonstrate enhanced viral binding breadth across 100 years of antigenic drifts and shifts of H1N1 viruses (Figure 3C), although to a lesser degree by subject (Figure S3F).

We next assessed whether polyreactive mAbs were more likely to cross-react with zoonotic influenza viruses. Over three-quarters of polyreactive $H1^+$ mAbs bound a swine H1N2 virus (A/swine/Mexico/AVX8/2011), as opposed to only 54% of non-polyreactive mAbs (Figure 3D; $p = 0.0017$). Similarly, 51% of polyreactive $H1^+$ mAbs recognized avian recombinant H5 in comparison with only 20% of non-polyreactive $H1^+$ mAbs (Figure 3E; $p < 0.0001$), and one-third of polyreactive $H3^+$ mAbs bound avian rH7, whereas not a single non-polyreactive $H3^+$ mAb bound rH7 (Figure 3F; $p = 0.0185$). Polyreactive mAbs binding the swine H1N2 virus and rH5 had appreciable affinity against these antigens (Figures S3G and S3H), indicating that cross-reactive polyreactive antibody responses would be the first line of defense against both swine and avian influenza viruses in adults previously exposed to human influenza viruses. H1N1-induced polyreactive mAbs had greater heterosubtypic binding to H3N2 viruses (Group 2; 45%) relative to non-polyreactive mAbs (23%; Figure 3G; $p < 0.0001$). Moreover, one-third of H1N1-binding polyreactive mAbs bound influenza B viruses of either the B/Yamagata/16/1988-like or B/Victoria/2/1987-like lineages (Figure 3H; $p = 0.0088$). Together, these data reveal that polyreactive mAbs have greater viral binding breadth, particularly to zoonotic viruses. These findings suggest that tolerance of polyreactivity in the antibody repertoire provides an important first line of defense against pandemic threat pathogens.

Polyreactivity Augments Viral Binding Affinity by Increasing Antibody Flexibility

We next evaluated whether polyreactivity qualitatively altered viral binding strength. Of the mAbs induced by first exposure to pH1N1 (Figure 2A), polyreactive mAbs had two-fold stronger apparent affinity for A/California/7/2009 pH1N1 virus in comparison with non-polyreactive mAbs (Figure 4A; $p = 0.0059$). Polyreactive head domain-binding mAbs had 17.5-fold stronger apparent affinity for the inducing pH1N1 virus relative to non-polyreactive head domain-binding mAbs (Figure 4B; $p = 0.0003$). Clonal expansions where one or multiple clonal members did or did not display polyreactivity revealed that the polyreactive member(s) always had stronger apparent affinity for its inducing strain relative to the non-polyreactive counterpart (Figure 4C; $p = 0.0039$). However, we did not observe any difference in the apparent association (K_a) or dissociation (K_d) of polyreactive and non-polyreactive clonal members by surface plasmon resonance (SPR; Figures S4A and S4B). Additionally, polyreactive binding strength positively correlated with viral binding strength (Figure 4D; Figure S4C; $p < 0.0001$), suggesting that polyreactivity could increase antigen-specific binding. Because polyreactive mAbs have higher apparent affinity for influenza viruses than do antigens used in the polyreactivity panel (Figure S1C), we assessed whether KLH could compete for polyreactive mAb binding against HA by using biolayer interferometry (BLI). SFV005 2G02, a strongly polyreactive mAb, could still bind HA after being saturated with KLH (Figure S4D) but could not bind to KLH after being saturated with HA (Figure S4E), indicating the preferential binding to HA. Polyreactive mAbs also constituted the 77% of neutralizing mAbs not targeting the HA head (Figure 4E), and a greater proportion of HA^+ HAI^- polyreactive mAbs were neutralizing in comparison with non-polyreactive mAbs (Figure S4F). There was no difference in the potency (IC_{50}) of neutralizing polyreactive and non-polyreactive mAbs targeting the stalk domain (Figure S4G), but we

CellPress
OPEN ACCESS

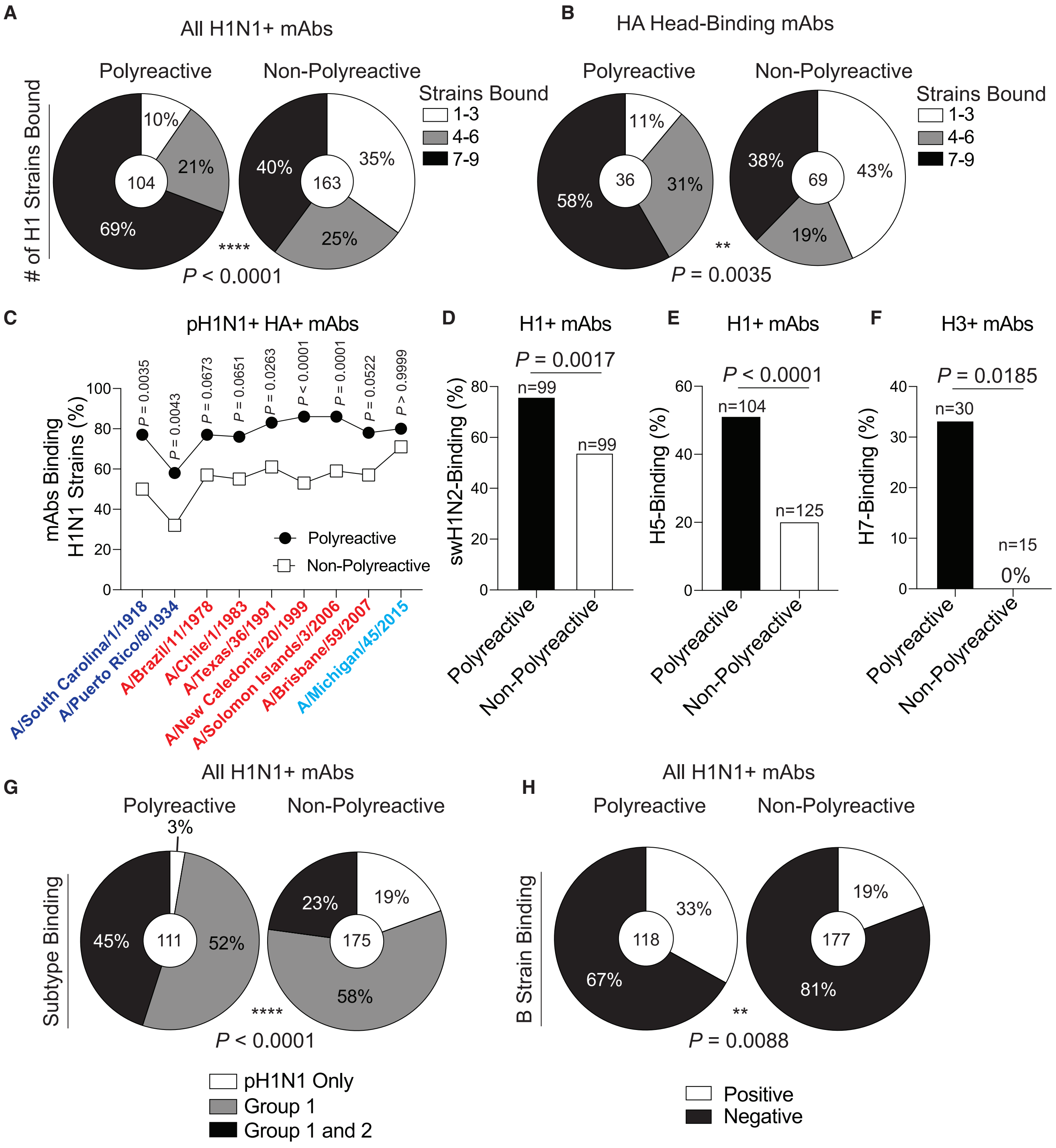

Figure 3. Polyreactive mAbs Have Broad Viral Binding Breadth

(A–C) Number of tested H1N1 strains bound by all (A) or head domain-binding (B) pH1N1$^+$ polyreactive and non-polyreactive mAbs.

(C) Proportion of polyreactive and non-polyreactive mAbs binding H1N1 viruses over time, with each symbol representing the proportion of mAbs binding each strain. Viruses are color coded based on antigenic similarity.

(D–F) Proportion of H1$^+$ mAbs binding A/swine/Mexico/AVX8/2011 H1N2 virus (D) or rH5 (E) and H3$^+$ mAbs binding rH7 (F).

(G and H) Proportion of H1N1$^+$ polyreactive and non-polyreactive mAbs binding Group 1 and Group 2 influenza viruses (G) or influenza B viruses (H).

For data in (A), (B), and (D)–(H), the numbers in the center of each pie graph or above each bar are the number of mAbs tested. Data in (A), (B), and (G) were analyzed by using chi-square tests, and data in (C)–(F) and (H) were analyzed by Fisher's exact test. See also Figure S3.

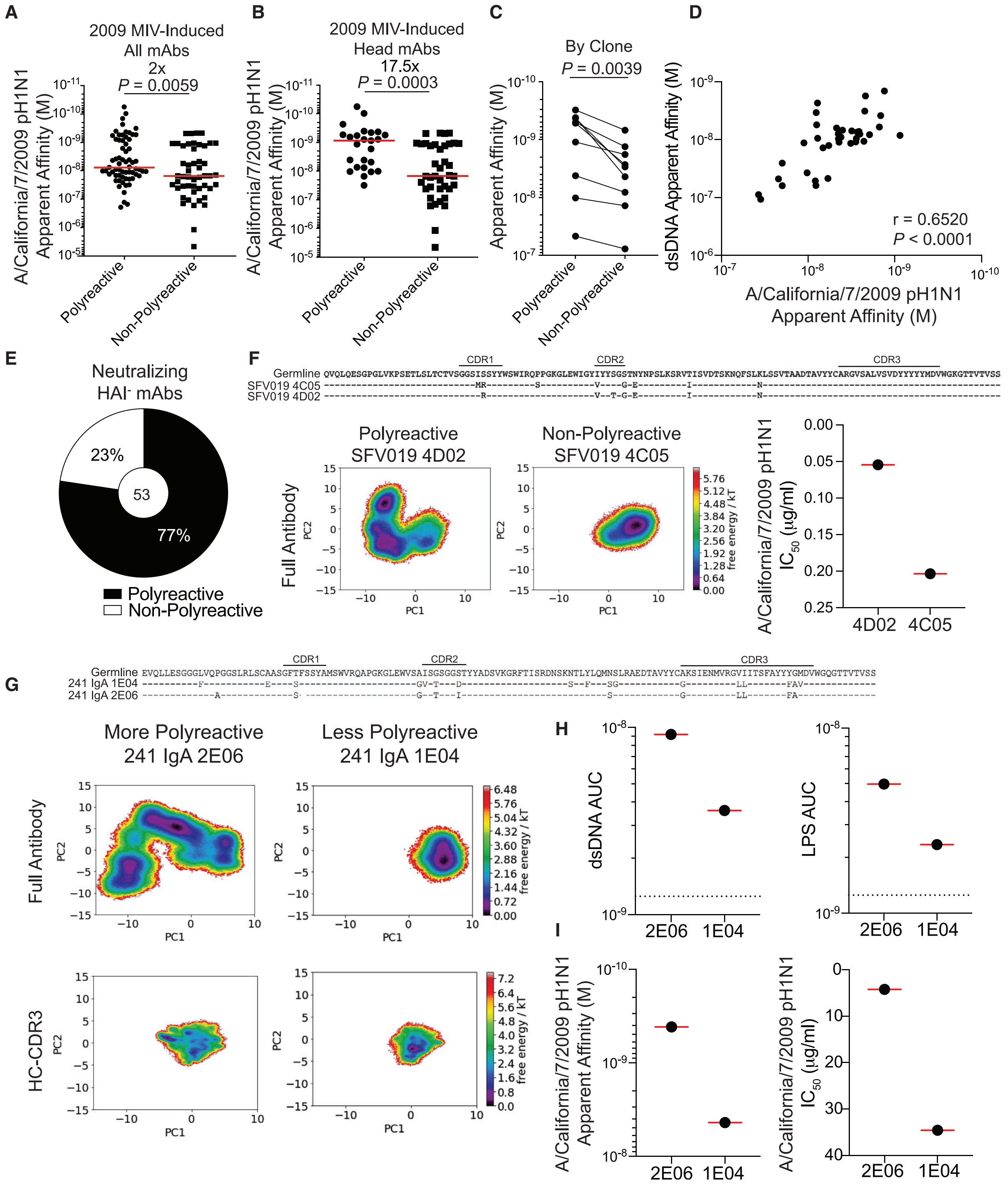

Figure 4. Polyreactivity Augments Viral Binding Apparent Affinity by Increasing Antibody Flexibility

(A and B) Apparent affinity (K_d) of all polyreactive (n = 65) and non-polyreactive (n = 49) mAbs (A) or head-binding polyreactive (n = 25) and non-polyreactive (n = 39) mAbs (B) induced by 2009 MIV binding to pH1N1 virus.

(C) Apparent affinity (K_d) of polyreactive and non-polyreactive mAbs from clonal families (n = 9). The line connects mAbs from the same clonal expansion, and each line is a different clonal family. For families with two or more polyreactive or non-polyreactive members, the median K_d is depicted.

(D) Spearman correlation of the apparent affinity (K_d) of polyreactive mAb binding to A/California/7/2009 virus and dsDNA (n = 37).

(legend continued on next page)

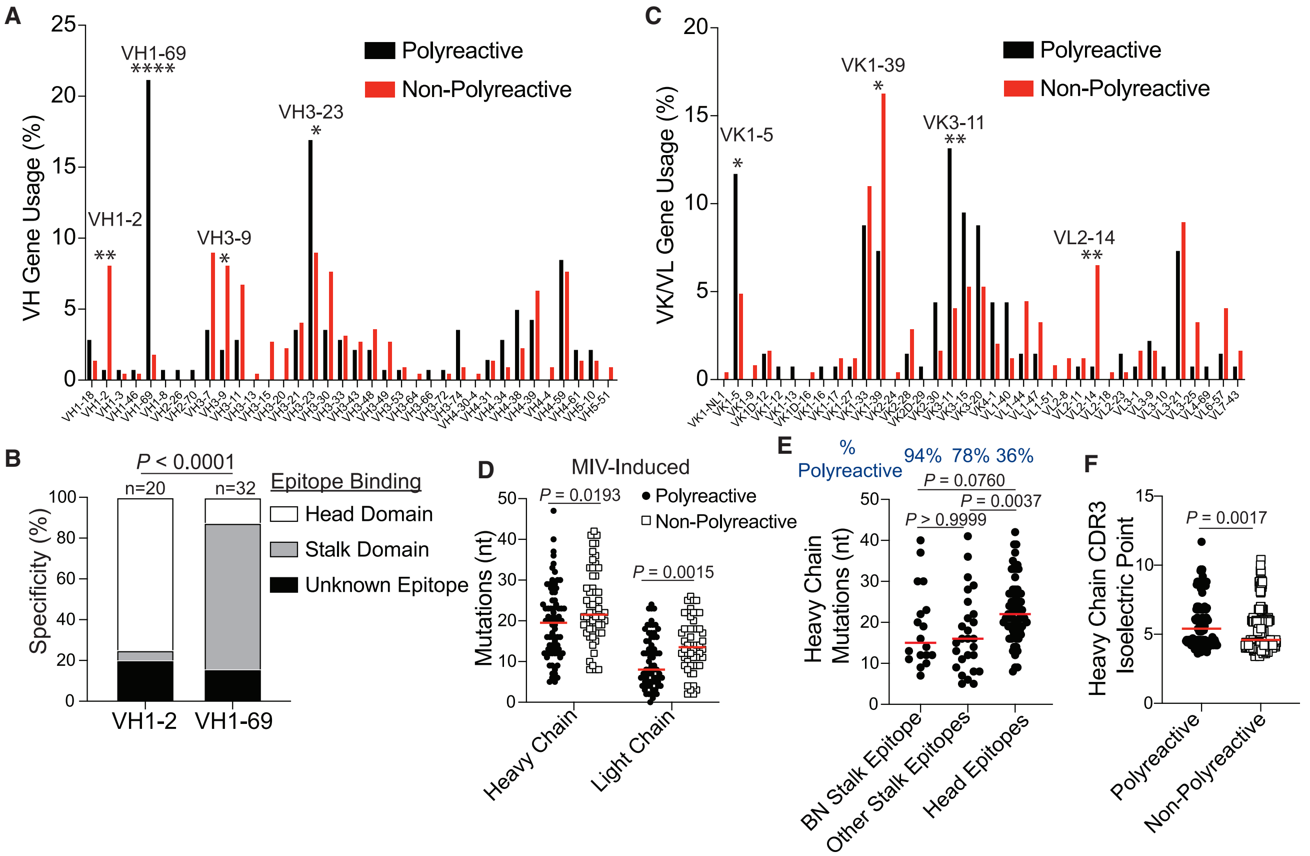

Figure 5. Characteristics of Polyreactive Antibody Sequences

(A) VH gene usage by polyreactive and non-polyreactive mAbs. Data are represented as the proportion of total polyreactive or non-polyreactive mAbs.

(B) Specific epitope targeting by mAbs utilizing VH1-2 and VH1-69 genes.

(C) VK or VL gene usage by polyreactive and non-polyreactive mAbs. Data are represented as the proportion of total polyreactive or non-polyreactive mAbs.

(D) Somatic hypermutations (nucleotide mutations) of heavy and light chains of polyreactive (heavy n = 74; light n = 69) and non-polyreactive (heavy n = 56; light n = 52) mAbs induced by the 2009 MIV.

(E) Somatic hypermutations of heavy chains of mAbs targeting the BN stalk epitope (n = 18), other epitopes on the stalk domain (n = 27), and epitopes on the head domain (n = 66) induced by the 2009 MIV.

(F) HC-CDR3 isoelectric point of polyreactive (n = 137) and non-polyreactive (n = 245) mAbs.

For data in (B), the number above each bar represents the number of mAbs tested. For panels (D)–(F), each symbol represents one mAb, and the red bar represents the median. Data in (A) and (C) were analyzed by Fisher's exact test, data in (B) were analyzed by using chi-square test, data in (D) and (F) were analyzed by unpaired non-parametric Mann-Whitney tests, and data in (E) were analyzed by a non-parametric Kruskal-Wallis test. See also Figure S5.

detected an increase in neutralization potency of polyreactive RBS- and lateral-patch-binding mAbs relative to non-polyreactive mAbs targeting the same epitopes (Figure S4H).

Polyreactivity can enhance antibody binding activity by increasing antibody variable region flexibility and promoting antibody heteroligation to a membrane proximal epitope and the viral membrane (Prigent et al., 2018; Mouquet et al., 2010). We evaluated the possibility of antibody flexibility by performing molecular dynamic (MD) simulations of polyreactive and non-polyreactive clonal members, as previously described (Fernández-Quintero et al., 2019). By first evaluating an RBS clone, we found the polyreactive member (SFV019 4D02) had shallower energy minima for structural rearrangement and displayed a broader confirmation ensemble (Figure 4F), both hallmarks of increased antibody flexibility. The polyreactive clonal member (4D02) demonstrated a near 4-fold increase in neutralization potency

(E) Proportion of neutralizing HAI^{-} mAbs that are polyreactive or non-polyreactive.

(F and G) MD simulations for clonal members SFV019 4C05 and 4D02 (F) and 241 IgA 1E04 and 2E06 (G). The right-hand panel of (F) is the neutralizing potency against A/California/7/2009 H1N1 (IC_{50}) of 4C05 and 4D02. Corresponding heavy-chain sequences are listed above simulations.

(H) Binding apparent affinity (AUC) of 241 IgA 1E04 and 2E06 to dsDNA and LPS.

(I) Apparent affinity and microneutralization potency (IC_{50}) of 241 IgA 1E04 and 2E06 against A/California/7/2009.

For data in panels (A), (B), (D), and (F)–(I), each symbol represents one mAb. For data in (E), the number in the center of the pie graph is the number of mAbs tested. Data in (A) and (B) were analyzed by unpaired non-parametric Mann-Whitney tests, and data in (C) were analyzed by using a paired non-parametric Wilcoxon matched-pairs signed rank test. See also Figure S4.

(Figure 4F), consistent with polyreactivity enhancing viral neutralization. We additionally performed MD simulations on a clone targeting the BN stalk epitope that demonstrated quantitative differences in polyreactivity, with 241 IgA 2E06 having stronger apparent affinity for dsDNA and LPS relative to 241 IgA 1E04 (Figures 4G and 4H). Both simulations of the full antibody and the HC-CDR3 of the more polyreactive 2E06 demonstrated reduced free-energy barriers and more conformational states relative to the less polyreactive 1E04 (Figure 4G). 2E06 demonstrated enhanced viral binding strength and neutralizing potency (Figure 4I), further suggesting that polyreactivity could play a critical role in antibody neutralization potency. Altogether, these data reveal that polyreactivity is associated with increased antibody flexibility within the variable regions, which in turn impacts viral binding strength, neutralization potency, and cross-reactivity.

Polyreactive B Cells Utilize V(D)J Genes Associated with Broadly Neutralizing Antibodies

We next investigated whether polyreactive mAbs used restricted V(D)J genes and had distinctive BCR features. Heavy chains of polyreactive mAbs used a wide range of V(D)J genes (Figure 5A; Figures S6A and S6B). However, VH1-69, a VH gene commonly used by stalk domain-binding mAbs, was almost exclusively used by polyreactive mAbs ($p < 0.0001$), whereas mAbs utilizing VH1-2 were nearly entirely non-polyreactive ($p = 0.0012$) and primarily targeted the HA head (Figures 5A and 5B). We further identified several light-chain biases of polyreactive and non-polyreactive mAbs (Figure 5C). Polyreactive and non-polyreactive mAbs generally used DH, JH, JK, and JL genes with similar frequencies (Figures S5A–S5C). Additionally, we identified polyreactive mAbs generated from plasmablasts induced by the 2009 MIV, which had not affinity-matured to the new pH1N1 virus, tended to harbor fewer nucleotide and amino acid mutations in both the heavy and light chains than did non-polyreactive mAbs induced by the 2009 MIV (Figure 5D; Figure S5D; heavy chain $p = 0.0193$; light chain $p = 0.0015$), suggesting that fewer mutations were required to acquire stronger affinity against a novel virus (Figure 4A). However, there were no differences in the number of mutations in polyreactive and non-polyreactive mAbs when assessing all cohorts tested (Figure S5E), likely as a result of the recruitment and affinity maturation of new naive B cells against the newly circulating pH1N1. Additionally, we found mAbs against the BN stalk epitope and other stalk domain epitopes, of which 95% and 76% were polyreactive, respectively, tended to harbor fewer mutations, compared to mAbs against the HA head, of which only 28% of mAbs were polyreactive (Figure 5E; BN stalk versus head epitopes $p = 0.0760$; other stalk epitopes versus head epitopes $p = 0.0037$). We found no difference in CDR3 length of polyreactive and non-polyreactive mAbs (Figure S5F), consistent with prior reports (Aguilera et al., 2001; Volpe and Kepler, 2009). Polyreactive CDR3s had higher isoelectric points relative to non-polyreactive CDR3s (Figure 5F; Figure S5G; $p = 0.0017$), supporting reports that polyreactive CDR3 sequences tend to possess basic residues, such as arginine and histidine (Zhang and Yeh, 1994; Shlomchik et al., 1987). Together, these data reveal that polyreactive mAbs utilize distinct V genes and harbor fewer mutations.

Polyreactive Naive B Cells Are Selected into the Broadly Neutralizing MBC Pool

It remains to be determined whether broadly neutralizing antibodies tend to acquire polyreactivity through affinity maturation or whether polyreactivity is inherent to the germline antibody expressed by naive B cells. To test this, we generated mAbs from B cells with germline antibody sequences that targeted the HA stalk domain. By using PBMCs from a chimeric HA (cHA) vaccine trial (Bernstein et al., 2020), we bait-sorted B cells with a cHA (cH5/1), in which the head domain was from an avian H5 virus and the stalk domain was from A/California/7/2009 H1, to preferentially pull out stalk-reactive B cells from which we could generate mAbs. Although we were able to detect B cells with germline or near-germline sequences (≤ 2 mutations; Figure S6A), we could not confirm these cells were naive B cells. 10% of the total naive B cell repertoire and 30% of the MBC repertoire were polyreactive (Figure 6A). In sharp contrast, 48% of germline B cells targeting the stalk domain were polyreactive (Figure 6A; $p = 0.0004$), indicating the frequency of polyreactive stalk domain-binding germline B cells was much higher than that of the general naive B cell repertoire. However, the proportion of polyreactive germline B cells targeting the stalk domain was less than the 69% and 88% of stalk domain-binding MBCs and plasmablasts, respectively (Figure 1D; Figure 6A; germline versus MBC $p = 0.1001$; germline versus plasmablast $p < 0.0001$), suggesting either polyreactive naive B cells were preferentially selected into the MBC compartment or non-polyreactive B cells acquired polyreactivity during affinity maturation. Polyreactive germline mAbs had similar affinity to recombinant H1 as non-polyreactive germline mAbs (Figure S6B), suggesting that initial HA-specific affinity did not play a role in the selection of polyreactive B cells into the MBC pool. To distinguish between the preferential selection of polyreactive naive B cells versus acquisition of polyreactivity during affinity maturation, we generated the germline version of affinity-matured polyreactive influenza virus-binding mAbs generated from plasmablasts, including mAbs targeting the head and stalk domains of HA. Nearly all germline mAbs had reduced binding to the pH1N1 virus (A/California/7/2009; $p = 0.0010$) and a pre-pH1N1 virus (A/Brisbane/59/2007; $p = 0.0005$) compared to the affinity-matured versions isolated (Figure 6B). However, all of the reverted germline mAbs were polyreactive (Figure 6C; Figure S6C), indicating polyreactive naive B cells were preferentially being selected into the affinity-matured MBC pool.

Although it was clear affinity maturation increased viral binding strength, it was unclear whether affinity maturation increased polyreactive binding (Figure 6D), with a 60:40 split of mAbs increasing polyreactive mAb binding with affinity maturation. When broken down by germline mAbs with high affinity or low affinity to A/California/7/2009 (Figure S6D), we found that the low-affinity germline mAbs that increased viral binding affinity during affinity maturation increased polyreactivity, whereas high-affinity germline mAbs reduced polyreactivity as they affinity-matured (Figure 6E). Notably, 80% of generated germline mAbs with high affinity to influenza viruses utilized VH1-69, in contrast to only 33% of generated low-affinity germline mAbs (Figure 6E). To investigate the discrepancy of affinity maturation on polyreactive mAb binding affinity, we performed MD simulations on the germline and affinity-matured versions of two mAbs (Figure S6E).

CellPress
OPEN ACCESS

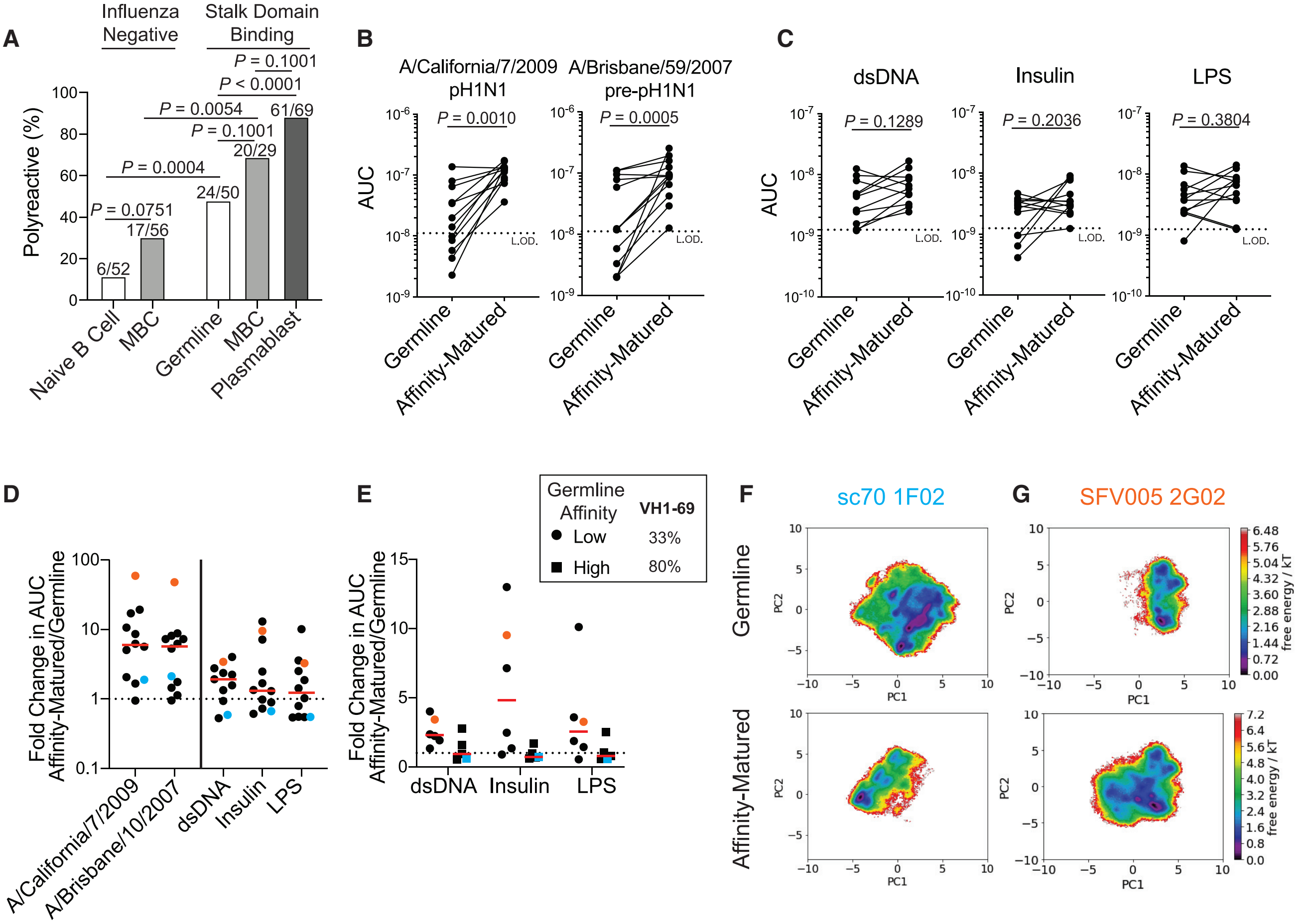

Figure 6. Polyreactivity Is Inherent to Naive B Cells Selected into the Broadly Neutralizing Response

(A) Proportion of mAbs generated from total naive B cells and MBCs and from influenza stalk domain-binding germline B cells, MBCs, and plasmablasts that were polyreactive.

(B and C) Binding AUC of polyreactive mAbs or the corresponding germline-reverted mAbs binding to A/California/7/2009 (n = 12) and A/Brisbane/59/2007 viruses (n = 13) (B) and dsDNA (n = 11), insulin (n = 12), and LPS (n = 12) (C). Each line connects the germline and affinity-matured version of the same mAb.

(D and E) Fold change in AUC of affinity-matured mAbs over AUC of germline mAbs of all tested mAbs (D) and further broken down by initial germline affinity for A/California/7/2009 virus (E). Cyan dots represent sc70 1F02, and orange dots represent SFV005 2G02.

(F and G) MD simulations of the HC-CDR3 sequences of germline and affinity-matured versions of mAbs sc70 1F02 (F) and SFV005 2G02 (G).

For (A), the numbers on top of individual bars is the number of polyreactive mAbs out of total mAbs tested from each cohort. For (D) and (E), each symbol represents one mAb, and the red bar represents the median. Data in (A) were analyzed by Fisher's exact test, and data in (B) and (C) were analyzed by paired non-parametric Wilcoxon matched-pairs signed rank tests. See also Figure S6.

We first evaluated sc70 1F02, which marginally increased affinity toward H1N1 viruses upon affinity maturation but reduced affinity for polyreactive antigens (Figures 6D and 6E; cyan dot or square). Notably, affinity-matured sc70 1F02 only utilizes residues present in the germline mAb to bind H5 (Nachbagauer et al., 2018), suggesting that the germline version of the mAb already has high affinity for Group 1 influenza viruses. By MD simulations, sc70 1F02 HC-CDR3 became more rigid with affinity maturation, indicating a higher free-energy barrier to change confirmations and fewer confirmations possible (Figure 6F). In contrast, SFV005 2G02 increased apparent affinity for H1N1 viruses and influenza virus-unrelated antigens upon affinity maturation (Figures 6D and 6E; orange dot). MD simulations revealed an increase in potential confirmations, as well as lower free-energy burdens to change confirmations, consistent with increased HC-CDR3 flexibility (Figure 6G). It should be noted that sc70 1F02 utilizes VH1-69, a well-characterized VH gene used by many stalk domain-binding antibodies (Wrammert et al., 2011). SFV005 2G02 utilizes VH1-18 and D3-9, which are used by many stalk domain-binding antibodies (Joyce et al., 2016; Wu et al., 2018). Together, these data reveal that naive B cells expressing polyreactive BCRs are selected into the broadly neutralizing MBC pool.

DISCUSSION

Our data revealed that mAbs targeting conserved epitopes on the HA stalk and head domains were polyreactive. Consistent with targeting conserved epitopes, polyreactive mAbs exhibited enhanced viral binding breadth to drifted and shifted

homosubtypic viruses and heterosubtypic viruses. Notably, the majority of mAbs cross-reactive to avian H5 and H7 antigens were polyreactive, suggesting that polyreactivity plays a critical role in antibody binding to imperfectly conserved epitopes on novel virus subtypes with pandemic potential. Cross-reactive polyreactive B cells induced by prior exposures to human influenza viruses could be the first line of defense against antigenically shifted and zoonotic influenza viruses, consistent with stalk antibodies being induced by novel influenza viruses (Andrews et al., 2015a; Li et al., 2012; Henry Dunand et al., 2016; Ellebedy et al., 2014; Thomson et al., 2012; Wrammert et al., 2011; Pica et al., 2012).

In addition to increased viral binding breadth, polyreactive mAbs had stronger apparent affinity for influenza viruses than did non-polyreactive mAbs. Notably, the context and antigen can change the role and characteristics of polyreactive mAbs. HIV-binding polyreactive mAbs are low affinity and non-neutralizing and derive from intestinal B cells (Planchais et al., 2019; Liao et al., 2011). However, HIV-binding polyreactive mAbs can become potently neutralizing against HIV through affinity maturation (Liao et al., 2011; Prigent et al., 2018). We found polyreactive members had increased antibody flexibility relative to non-polyreactive clonal members, which was associated with increased apparent affinity and neutralization potency. Mutations that increased HIV viral binding and neutralization breadth often led to antibody polyreactivity (Prigent et al., 2018), suggesting that polyreactivity is selected to increase apparent affinity and neutralization capabilities. Together, these data show polyreactivity is advantageous for influenza virus antibody binding affinity and illustrate a clear selective advantage for having immune tolerance of polyreactivity in the B cell repertoire.

Polyreactive mAbs tend to use V(D)J genes associated with broadly neutralizing mAbs, such as VH1-69 and VH3-23 (Wrammert et al., 2011; De Marco et al., 2012). Because VH1-69 utilizing antibodies commonly target the HA stalk domain and are polyreactive, polyreactivity could account for the overrepresentation of stalk domain-binding antibodies using this particular VH gene. However, not all VH1-69-utilizing antibodies target the stalk domain and are polyreactive, as VH1-69-utilizing head-binding antibodies are not polyreactive (Andrews et al., 2015a). However, no particular V(D)J gene was exclusively associated with polyreactivity, suggesting that the factors that allow for polyreactivity are more complex than the V(D)J genes used by any one antibody. Biochemically, mAbs that are polyreactive often have long, flexible, and hydrophobic or positively charged CDRs (Prigent et al., 2018; Fernández-Quintero et al., 2019; Deng and Notkins, 2000). However, polyreactivity could come at an energetic cost, because increased antibody flexibility is associated with unfavorable entropy and enthalpy measurements (Prigent et al., 2018). Despite unfavorable thermodynamic binding, polyreactivity is selected for within the broadly neutralizing antibody pool against HIV and influenza viruses (Andrews et al., 2015a; Chen et al., 2014), suggesting that energetic costs are overcome to provide broad neutralizing breadth. Furthermore, the specific polyreactive interactions that enhance affinity remain unknown, and further investigation into what specific residues or motifs confer polyreactivity during pathogen-induced antibody responses is necessary to address these important questions. Traditionally, polyreactive "natural" antibodies were thought to solely be the product of innate-like B cells (Capolunghi et al., 2008; Chou et al., 2009). Our data demonstrate that polyreactive mAbs isolated in response to influenza virus exposure also arise from MBCs that are affinity-matured, isotype switched, and show true antigen specificity and function.

Our data demonstrated that polyreactivity is inherent to responding B cells rather than acquired during affinity maturation. Polyreactivity is also a common feature in the selection of B cell lineages with broadly neutralizing potential against HIV (Roskin et al., 2020), suggesting that germline targeting of polyreactive B cell clones could be critical for the generation of broadly neutralizing antibodies. Our data also showed that affinity maturation did not necessarily increase polyreactivity and appeared to be dependent on the initial affinity for antigen. Naive B cells with high affinity are proposed to become more rigid with affinity maturation, whereas antibodies with low affinity for their antigen become more flexible (Ovchinnikov et al., 2018). Notably, VH1-69-utilizing mAbs, including sc70 1F02, require limited affinity maturation to prompt strong affinity to influenza viruses (Sangesland et al., 2019; Pappas et al., 2014). Additionally, we found that polyreactive mAbs induced by the monovalent pH1N1 vaccine had fewer somatic hypermutations than did non-polyreactive mAbs, implying that polyreactive mAbs require fewer mutations to recognize a divergent influenza virus and undergo fewer rounds of selection within the germinal center. Alternatively, polyreactivity could attenuate polyreactive B cell affinity maturation, resulting in earlier exit from the GC and fewer mutations.

High-affinity polyreactive mAbs can cause autoimmune pathologies (Zhang et al., 2009). Despite this, polyreactive mAbs are not increased after immunization in individuals with autoimmune diseases (Kaur et al., 2015), and nearly every antigenic exposure induces polyreactive antibody responses (Dimitrov et al., 2013), suggesting that polyreactivity is a general feature of humoral immunity in healthy individuals. However, B cells with polyreactive tendencies tend to be counter-selected during B cell development (Wardemann et al., 2003; Zhang et al., 2016) and can become anergic naive B cells (Duty et al., 2009; Smith et al., 2015) and atypical MBCs (Muellenbeck et al., 2013). Further research is needed to understand whether polyreactive broadly neutralizing B cells against influenza viruses are counter-selected or become anergic. Notably, breaking B cell tolerance or blocking immune checkpoints allows for the selection of high-affinity B cell clones and broadly neutralizing B cells (Kaur et al., 2015; Schroeder et al., 2017; Bradley et al., 2020), suggesting that the precursors of B cells against conserved epitopes are limited by clonal deletion or anergy. Mice with a genetically targeted introduction of a polyreactive HIV-binding BCR counter-select these polyreactive B cells (Verkoczy et al., 2010), indicating B cell tolerance limits polyreactive pathogen-binding antibody responses. Together, our data and past studies suggest careful consideration will be needed to induce a robust antibody response against conserved epitopes.

Limitations of Study

The data in the study indicate that polyreactivity is associated with increased viral binding breadth and are preferentially recalled by antigenically novel influenza viruses, but whether polyreactivity is required for broad viral binding is not known.

Moreover, it is unknown whether and how antibody flexibility increases viral binding strength and breadth. Although our study finds clear correlative data relating to the role of polyreactivity for viral binding breadth, affinity, and antibody flexibility, it is not known what features of antibodies mediate polyreactivity. High-resolution structures of polyreactive and non-polyreactive clonal members binding HA could help identify the role of antibody flexibility in the context of viral binding and the potential contacts leading to polyreactive antibody binding.

STAR★METHODS

Detailed methods are provided in the online version of this paper and include the following:

- KEY RESOURCES TABLE
- RESOURCE AVAILABILITY
  - Lead Contact
  - Materials Availability
  - Data and Code Availability
- EXPERIMENTAL MODEL AND SUBJECT DETAILS
  - Human Materials
  - Cell Lines
- METHOD DETAILS
  - Monoclonal antibody production
  - Viruses and recombinant proteins
  - Antigen-Specific ELISA
  - Polyreactive ELISA
  - Microneutralization and Hemagglutination Inhibition Assays (HAI)
  - Molecular Dynamics Simulations
  - HA models
  - Size Exclusion Chromatography
  - Biolayer Interferometry (BLI)
  - Surface Plasmon Resonance (SPR)
- QUANTIFICATION AND STATISTICAL ANALYSIS

SUPPLEMENTAL INFORMATION

Supplemental Information can be found online at https://doi.org/10.1016/j.immuni.2020.10.005.

ACKNOWLEDGMENTS

This project was funded in part by the National Institute of Allergy and Infectious Diseases (NIAID); National Institutes of Health grant numbers U19AI082724 (P.C.W.), U19AI109946 (P.C.W.), U19AI057266 (P.C.W.), P01 AI097092 (P.P.), R01AI145870-01 (P.P.), and T32AI007244-36 (J.H.); and the NIAID Centers of Excellence for Influenza Research and Surveillance (CEIRS) grant number HHSN272201400005C (P.C.W.) and HHSN272201400008C (L.C., F.K., A.G.-S., P.P.). This work was also partially supported by the NIAID Collaborative Influenza Vaccine Innovation Centers (CIVIC; 75N93019C00051, F.K., A.G.-S., P.P., A.B.W., P.C.W.). Work related to MD simulations were funded by the Austrian Science Fund grant number P30565 (K.R.L.). We are thankful to the teams at PATH, GlaxoSmithKline, Cincinnati Children's Hospital Medical Center, and Duke University for their participation in the chimeric HA vaccine trial NCT03300050, which was funded by the Bill and Melinda Gates Foundation (OPP1084518). This work is based on research funded in part by the Bill & Melinda Gates Foundation. The findings and conclusions contained within are those of the authors and do not necessarily reflect positions or policies of the Gates Foundation. We thank Sarah Andrews, Jens Wrammert, and Rafi Ahmed for participation in acquiring samples from 2009 MIV and 2010 TIV studies. We thank Erin Adams, Albert Bendelac, Steven Erickson, Christopher Boughter, and Nancy D. Pomarici for fruitful discussion and their support in this project. We also thank Elena Solomaha in the University of Chicago Biophysics Core Facility for her assistance.

AUTHOR CONTRIBUTIONS

J.J.G. designed and performed experiments, analyzed the data, and wrote the manuscript. L.Y.L. bait-sorted and single-cell sequenced B cells and generated monoclonal antibodies. M.L.F.-Q. and K.R.L. performed MD simulations. J.H. and A.B.W. generated HA structures and assisted with epitope identification. N.J.H., D.J.B., and M.E.T. performed polyreactive ELISAs and virus-specific ELISAs. C.H., K.E.N., H.L.D., M.E.T., C.T.S., A-K.E.P., and Y.-Q.C. sorted plasmablasts and generated mAbs for the vaccination and infection cohorts. L.L. analyzed sequencing data and helped perform statistical analyses. M.T.B. performed size exclusion chromatography. O.S. performed microneutralization assays and edited the manuscript. H.A.U. helped perform SPR and BLI experiments. D.J.B. helped analyze antibody repertoire data. N.-Y.Z. purified influenza viruses and provided materials. M.H. performed mAb cloning. S.T.H.L., P.P., F.K., and L.C. provided recombinant proteins and assistance with epitope identification. F.K., P.P., A.G.-S., and R.N. designed and orchestrated cHA vaccination trial. P.C.W. supervised the work and wrote the manuscript.

DECLARATION OF INTERESTS

The Icahn School of Medicine at Mount Sinai has submitted patent applications on universal influenza virus vaccines naming R.N., A.G.-S., P.P., and F.K. as inventors.

Received: May 20, 2020
Revised: September 14, 2020
Accepted: October 7, 2020
Published: October 22, 2020

REFERENCES

Adelman, S.A., and Doll, J.D. (1975). Generalized Langevin equation approach for atom/solid-surface scattering: Inelastic studies. J. Chem. Phys. *64*, 2375.

Aguilera, I., Melero, J., Nuñez-Roldan, A., and Sanchez, B. (2001). Molecular structure of eight human autoreactive monoclonal antibodies. Immunology *102*, 273–280.

Andrews, S.F., Huang, Y., Kaur, K., Popova, L.I., Ho, I.Y., Pauli, N.T., Henry Dunand, C.J., Taylor, W.M., Lim, S., Huang, M., et al. (2015a). Immune history profoundly affects broadly protective B cell responses to influenza. Sci. Transl. Med. *7*, 316ra192.

Andrews, S.F., Kaur, K., Pauli, N.T., Huang, M., Huang, Y., and Wilson, P.C. (2015b). High preexisting serological antibody levels correlate with diversification of the influenza vaccine response. J. Virol. *89*, 3308–3317.

Angeletti, D., Kosik, I., Santos, J.J.S., Yewdell, W.T., Boudreau, C.M., Mallajosyula, V.V.A., Mankowski, M.C., Chambers, M., Prabhakaran, M., Hickman, H.D., et al. (2019). Outflanking immunodominance to target subdominant broadly neutralizing epitopes. Proc. Natl. Acad. Sci. USA *116*, 13474–13479.

Bajic, G., van der Poel, C.E., Kuraoka, M., Schmidt, A.G., Carroll, M.C., Kelsoe, G., and Harrison, S.C. (2019). Autoreactivity profiles of influenza hemagglutinin broadly neutralizing antibodies. Sci. Rep. *9*, 3492.

Berendsen, H., Postma, J.P.M., van Gunsteren, W.F., DiNola, A., and Haak, J.R. (1984). Molecular-Dynamics with Coupling to An External Bath. J. Chem. Phys. *81*, 3684.

Bernstein, D.I., Guptill, J., Naficy, A., Nachbagauer, R., Berlanda-Scorza, F., Feser, J., Wilson, P.C., Solórzano, A., Van der Wielen, M., Walter, E.B., et al. (2020). Immunogenicity of chimeric haemagglutinin-based, universal influenza virus vaccine candidates: interim results of a randomised, placebo-controlled, phase 1 clinical trial. Lancet Infect. Dis. *20*, 80–91.

Bradley, T., Kuraoka, M., Yeh, C.H., Tian, M., Chen, H., Cain, D.W., Chen, X., Cheng, C., Ellebedy, A.H., Parks, R., et al. (2020). Immune checkpoint modulation enhances HIV-1 antibody induction. Nat. Commun. *11*, 948.

Bunker, J.J., Erickson, S.A., Flynn, T.M., Henry, C., Koval, J.C., Meisel, M., Jabri, B., Antonopoulos, D.A., Wilson, P.C., and Bendelac, A. (2017). Natural polyreactive IgA antibodies coat the intestinal microbiota. Science *358*, 358.

Capolunghi, F., Cascioli, S., Giorda, E., Rosado, M.M., Plebani, A., Auriti, C., Seganti, G., Zuntini, R., Ferrari, S., Cagliuso, M., et al. (2008). CpG drives human transitional B cells to terminal differentiation and production of natural antibodies. J. Immunol. *180*, 800–808.

Chen, J., Frey, G., Peng, H., Rits-Volloch, S., Garrity, J., Seaman, M.S., and Chen, B. (2014). Mechanism of HIV-1 neutralization by antibodies targeting a membrane-proximal region of gp41. J. Virol. *88*, 1249–1258.

Chen, Y.Q., Wohlbold, T.J., Zheng, N.Y., Huang, M., Huang, Y., Neu, K.E., Lee, J., Wan, H., Rojas, K.T., Kirkpatrick, E., et al. (2018). Influenza Infection in Humans Induces Broadly Cross-Reactive and Protective Neuraminidase-Reactive Antibodies. Cell *173*, 417–429.e10, e10.

Chothia, C., and Lesk, A.M. (1987). Canonical structures for the hypervariable regions of immunoglobulins. J. Mol. Biol. *196*, 901–917.

Chothia, C., Lesk, A.M., Tramontano, A., Levitt, M., Smith-Gill, S.J., Air, G., Sheriff, S., Padlan, E.A., Davies, D., Tulip, W.R., et al. (1989). Conformations of immunoglobulin hypervariable regions. Nature *342*, 877–883.

Chou, M.Y., Fogelstrand, L., Hartvigsen, K., Hansen, L.F., Woelkers, D., Shaw, P.X., Choi, J., Perkmann, T., Bäckhed, F., Miller, Y.I., et al. (2009). Oxidation-specific epitopes are dominant targets of innate natural antibodies in mice and humans. J. Clin. Invest. *119*, 1335–1349.

Corti, D., Voss, J., Gamblin, S.J., Codoni, G., Macagno, A., Jarrossay, D., Vachieri, S.G., Pinna, D., Minola, A., Vanzetta, F., et al. (2011). A neutralizing antibody selected from plasma cells that binds to group 1 and group 2 influenza A hemagglutinins. Science *333*, 850–856.

De Marco, D., Clementi, N., Mancini, N., Solforosi, L., Moreno, G.J., Sun, X., Tumpey, T.M., Gubareva, L.V., Mishin, V., Clementi, M., and Burioni, R. (2012). A non-VH1-69 heterosubtypic neutralizing human monoclonal antibody protects mice against H1N1 and H5N1 viruses. PLoS ONE *7*, e34415.

Deng, Y.J., and Notkins, A.L. (2000). Molecular determinants of polyreactive antibody binding: HCDR3 and cyclic peptides. Clin. Exp. Immunol. *119*, 69–76.

Dennison, S.M., Anasti, K., Scearce, R.M., Sutherland, L., Parks, R., Xia, S.M., Liao, H.X., Gorny, M.K., Zolla-Pazner, S., Haynes, B.F., and Alam, S.M. (2011). Nonneutralizing HIV-1 gp41 envelope cluster II human monoclonal antibodies show polyreactivity for binding to phospholipids and protein autoantigens. J. Virol. *85*, 1340–1347.

Dimitrov, J.D., Planchais, C., Roumenina, L.T., Vassilev, T.L., Kaveri, S.V., and Lacroix-Desmazes, S. (2013). Antibody polyreactivity in health and disease: statu variabilis. J. Immunol. *191*, 993–999.

Dreyfus, C., Laursen, N.S., Kwaks, T., Zuijdgeest, D., Khayat, R., Ekiert, D.C., Lee, J.H., Metlagel, Z., Bujny, M.V., Jongeneelen, M., et al. (2012). Highly conserved protective epitopes on influenza B viruses. Science *337*, 1343–1348.

Duty, J.A., Szodoray, P., Zheng, N.Y., Koelsch, K.A., Zhang, Q., Swiatkowski, M., Mathias, M., Garman, L., Helms, C., Nakken, B., et al. (2009). Functional anergy in a subpopulation of naive B cells from healthy humans that express autoreactive immunoglobulin receptors. J. Exp. Med. *206*, 139–151.

Ekiert, D.C., Bhabha, G., Elsliger, M.A., Friesen, R.H., Jongeneelen, M., Throsby, M., Goudsmit, J., and Wilson, I.A. (2009). Antibody recognition of a highly conserved influenza virus epitope. Science *324*, 246–251.

Ekiert, D.C., Kashyap, A.K., Steel, J., Rubrum, A., Bhabha, G., Khayat, R., Lee, J.H., Dillon, M.A., O'Neil, R.E., Faynboym, A.M., et al. (2012). Cross-neutralization of influenza A viruses mediated by a single antibody loop. Nature *489*, 526–532.

Ellebedy, A.H., Krammer, F., Li, G.M., Miller, M.S., Chiu, C., Wrammert, J., Chang, C.Y., Davis, C.W., McCausland, M., Elbein, R., et al. (2014). Induction of broadly cross-reactive antibody responses to the influenza HA stem region following H5N1 vaccination in humans. Proc. Natl. Acad. Sci. USA *111*, 13133–13138.

Fernández-Quintero, M.L., Loeffler, J.R., Kraml, J., Kahler, U., Kamenik, A.S., and Liedl, K.R. (2019). Characterizing the Diversity of the CDR-H3 Loop Conformational Ensembles in Relationship to Antibody Binding Properties. Front. Immunol. *9*, 3065.

Guthmiller, J.J., Dugan, H.L., Neu, K.E., Lan, L.Y., and Wilson, P.C. (2019). An Efficient Method to Generate Monoclonal Antibodies from Human B Cells. Methods Mol. Biol. *1904*, 109–145.

Hamelberg, D., Mongan, J., and McCammon, J.A. (2004). Accelerated molecular dynamics: a promising and efficient simulation method for biomolecules. J. Chem. Phys. *120*, 11919–11929.

Hamelberg, D., de Oliveira, C.A., and McCammon, J.A. (2007). Sampling of slow diffusive conformational transitions with accelerated molecular dynamics. J. Chem. Phys. *127*, 155102.

Haynes, B.F., Fleming, J., St Clair, E.W., Katinger, H., Stiegler, G., Kunert, R., Robinson, J., Scearce, R.M., Plonk, K., Staats, H.F., et al. (2005). Cardiolipin polyspecific autoreactivity in two broadly neutralizing HIV-1 antibodies. Science *308*, 1906–1908.

Henry, C., Zheng, N.Y., Huang, M., Cabanov, A., Rojas, K.T., Kaur, K., Andrews, S.F., Palm, A.E., Chen, Y.Q., Li, Y., et al. (2019). Influenza Virus Vaccination Elicits Poorly Adapted B Cell Responses in Elderly Individuals. Cell Host Microbe *25*, 357–366.e6, e6.

Henry Dunand, C.J., Leon, P.E., Kaur, K., Tan, G.S., Zheng, N.Y., Andrews, S., Huang, M., Qu, X., Huang, Y., Salgado-Ferrer, M., et al. (2015). Preexisting human antibodies neutralize recently emerged H7N9 influenza strains. J. Clin. Invest. *125*, 1255–1268.

Henry Dunand, C.J., Leon, P.E., Huang, M., Choi, A., Chromikova, V., Ho, I.Y., Tan, G.S., Cruz, J., Hirsh, A., Zheng, N.Y., et al. (2016). Both Neutralizing and Non-Neutralizing Human H7N9 Influenza Vaccine-Induced Monoclonal Antibodies Confer Protection. Cell Host Microbe *19*, 800–813.

Impagliazzo, A., Milder, F., Kuipers, H., Wagner, M.V., Zhu, X., Hoffman, R.M., van Meersbergen, R., Huizingh, J., Wanningen, P., Verspuij, J., et al. (2015). A stable trimeric influenza hemagglutinin stem as a broadly protective immunogen. Science *349*, 1301–1306.

Jorgensen, W.L., Chandrasekhar, J., Madura, J.D., Impey, R.W., and Klein, M.L. (1983). Comparison of simple potential functions for simulating liquid water. J. Chem. Phys. *79*, 926–935.

Joyce, M.G., Wheatley, A.K., Thomas, P.V., Chuang, G.Y., Soto, C., Bailer, R.T., Druz, A., Georgiev, I.S., Gillespie, R.A., Kanekiyo, M., et al.; NISC Comparative Sequencing Program (2016). Vaccine-Induced Antibodies that Neutralize Group 1 and Group 2 Influenza A Viruses. Cell *166*, 609–623.

Kaur, K., Zheng, N.Y., Smith, K., Huang, M., Li, L., Pauli, N.T., Henry Dunand, C.J., Lee, J.H., Morrissey, M., Wu, Y., et al. (2015). High Affinity Antibodies against Influenza Characterize the Plasmablast Response in SLE Patients After Vaccination. PLoS ONE *10*, e0125618.

Koelsch, K., Zheng, N.Y., Zhang, Q., Duty, A., Helms, C., Mathias, M.D., Jared, M., Smith, K., Capra, J.D., and Wilson, P.C. (2007). Mature B cells class switched to IgD are autoreactive in healthy individuals. J. Clin. Invest. *117*, 1558–1565.

Labute, P. (2009). Protonate3D: assignment of ionization states and hydrogen coordinates to macromolecular structures. Proteins *75*, 187–205.

Li, G.M., Chiu, C., Wrammert, J., McCausland, M., Andrews, S.F., Zheng, N.Y., Lee, J.H., Huang, M., Qu, X., Edupuganti, S., et al. (2012). Pandemic H1N1 influenza vaccine induces a recall response in humans that favors broadly cross-reactive memory B cells. Proc. Natl. Acad. Sci. USA *109*, 9047–9052.

Liao, H.X., Chen, X., Munshaw, S., Zhang, R., Marshall, D.J., Vandergrift, N., Whitesides, J.F., Lu, X., Yu, J.S., Hwang, K.K., et al. (2011). Initial antibodies binding to HIV-1 gp41 in acutely infected subjects are polyreactive and highly mutated. J. Exp. Med. *208*, 2237–2249.

Liu, M., Yang, G., Wiehe, K., Nicely, N.I., Vandergrift, N.A., Rountree, W., Bonsignori, M., Alam, S.M., Gao, J., Haynes, B.F., and Kelsoe, G. (2015). Polyreactivity and autoreactivity among HIV-1 antibodies. J. Virol. *89*, 784–798.

Maier, J.A., Martinez, C., Kasavajhala, K., Wickstrom, L., Hauser, K.E., and Simmerling, C. (2015). ff14SB: Improving the Accuracy of Protein Side Chain

CellPress
OPEN ACCESS

and Backbone Parameters from ff99SB. J. Chem. Theory Comput. *11*, 3696–3713.

Mena, I., Nelson, M.I., Quezada-Monroy, F., Dutta, J., Cortes-Fernández, R., Lara-Puente, J.H., Castro-Peralta, F., Cunha, L.F., Trovão, N.S., Lozano-Dubernard, B., et al. (2016). Origins of the 2009 H1N1 influenza pandemic in swine in Mexico. eLife *5*, 5.

Miao, Y., Sinko, W., Pierce, L., Bucher, D., Walker, R.C., and McCammon, J.A. (2014). Improved Reweighting of Accelerated Molecular Dynamics Simulations for Free Energy Calculation. J. Chem. Theory Comput. *10*, 2677–2689.

Millman, K.J., and Aivazis, M. (2011). Python for Scientists and Engineers. Comput. Sci. Eng. *13*, 9–12.

Miyamoto, S., and Kollman, P.J. (1992). Settle: An analytical version of the SHAKE and RATTLE algorithm for rigid water models. J. Comput. Chem. *13*, 952–962.

Mouquet, H., Scheid, J.F., Zoller, M.J., Krogsgaard, M., Ott, R.G., Shukair, S., Artyomov, M.N., Pietzsch, J., Connors, M., Pereyra, F., et al. (2010). Polyreactivity increases the apparent affinity of anti-HIV antibodies by heteroligation. Nature *467*, 591–595.

Muellenbeck, M.F., Ueberheide, B., Amulic, B., Epp, A., Fenyo, D., Busse, C.E., Esen, M., Theisen, M., Mordmüller, B., and Wardemann, H. (2013). Atypical and classical memory B cells produce Plasmodium falciparum neutralizing antibodies. J. Exp. Med. *210*, 389–399.

Nachbagauer, R., Shore, D., Yang, H., Johnson, S.K., Gabbard, J.D., Tompkins, S.M., Wrammert, J., Wilson, P.C., Stevens, J., Ahmed, R., et al. (2018). Broadly Reactive Human Monoclonal Antibodies Elicited following Pandemic H1N1 Influenza Virus Exposure Protect Mice against Highly Pathogenic H5N1 Challenge. J. Virol. *92*, 92.

Neu, K.E., Guthmiller, J.J., Huang, M., La, J., Vieira, M.C., Kim, K., Zheng, N.Y., Cortese, M., Tepora, M.E., Hamel, N.J., et al. (2019). Spec-seq unveils transcriptional subpopulations of antibody-secreting cells following influenza vaccination. J. Clin. Invest. *129*, 93–105.

Ng, S., Nachbagauer, R., Balmaseda, A., Stadlbauer, D., Ojeda, S., Patel, M., Rajabhathor, A., Lopez, R., Guglia, A.F., Sanchez, N., et al. (2019). Novel correlates of protection against pandemic H1N1 influenza A virus infection. Nat. Med. *25*, 962–967.

Ovchinnikov, V., Louveau, J.E., Barton, J.P., Karplus, M., and Chakraborty, A.K. (2018). Role of framework mutations and antibody flexibility in the evolution of broadly neutralizing antibodies. eLife *7*, 7.

Pappas, L., Foglierini, M., Piccoli, L., Kallewaard, N.L., Turrini, F., Silacci, C., Fernandez-Rodriguez, B., Agatic, G., Giacchetto-Sasselli, I., Pellicciotta, G., et al. (2014). Rapid development of broadly influenza neutralizing antibodies through redundant mutations. Nature *516*, 418–422.

Paules, C.I., Marston, H.D., Eisinger, R.W., Baltimore, D., and Fauci, A.S. (2017). The Pathway to a Universal Influenza Vaccine. Immunity *47*, 599–603.

Pettersen, E.F., Goddard, T.D., Huang, C.C., Couch, G.S., Greenblatt, D.M., Meng, E.C., and Ferrin, T.E. (2004). UCSF Chimera–a visualization system for exploratory research and analysis. J. Comput. Chem. *25*, 1605–1612.

Pica, N., Hai, R., Krammer, F., Wang, T.T., Maamary, J., Eggink, D., Tan, G.S., Krause, J.C., Moran, T., Stein, C.R., et al. (2012). Hemagglutinin stalk antibodies elicited by the 2009 pandemic influenza virus as a mechanism for the extinction of seasonal H1N1 viruses. Proc. Natl. Acad. Sci. USA *109*, 2573–2578.

Planchais, C., Kök, A., Kanyavuz, A., Lorin, V., Bruel, T., Guivel-Benhassine, F., Rollenske, T., Prigent, J., Hieu, T., Prazuck, T., et al. (2019). HIV-1 Envelope Recognition by Polyreactive and Cross-Reactive Intestinal B Cells. Cell Rep. *27*, 572–585.e7, e7.

Prigent, J., Jarossay, A., Planchais, C., Eden, C., Dufloo, J., Kök, A., Lorin, V., Vratskikh, O., Couderc, T., Bruel, T., et al. (2018). Conformational Plasticity in Broadly Neutralizing HIV-1 Antibodies Triggers Polyreactivity. Cell Rep. *23*, 2568–2581.

Raymond, D.D., Bajic, G., Ferdman, J., Suphaphiphat, P., Settembre, E.C., Moody, M.A., Schmidt, A.G., and Harrison, S.C. (2018). Conserved epitope on influenza-virus hemagglutinin head defined by a vaccine-induced antibody. Proc. Natl. Acad. Sci. USA *115*, 168–173.

Roe, D.R., and Cheatham, T.E., 3rd (2013). PTRAJ and CPPTRAJ: Software for Processing and Analysis of Molecular Dynamics Trajectory Data. J. Chem. Theory Comput. *9*, 3084–3095.

Roskin, K.M., Jackson, K.J.L., Lee, J.Y., Hoh, R.A., Joshi, S.A., Hwang, K.K., Bonsignori, M., Pedroza-Pacheco, I., Liao, H.X., Moody, M.A., et al. (2020). Aberrant B cell repertoire selection associated with HIV neutralizing antibody breadth. Nat. Immunol. *21*, 199–209.

Salomon-Ferrer, R., Götz, A.W., Poole, D., Le Grand, S., and Walker, R.C. (2013). Routine Microsecond Molecular Dynamics Simulations with AMBER on GPUs. 2. Explicit Solvent Particle Mesh Ewald. J. Chem. Theory Comput. *9*, 3878–3888.

Sangesland, M., Ronsard, L., Kazer, S.W., Bals, J., Boyoglu-Barnum, S., Yousif, A.S., Barnes, R., Feldman, J., Quirindongo-Crespo, M., McTamney, P.M., et al. (2019). Germline-Encoded Affinity for Cognate Antigen Enables Vaccine Amplification of a Human Broadly Neutralizing Response against Influenza Virus. Immunity *51*, 735–749.e8, e8.

Scheid, J.F., Mouquet, H., Feldhahn, N., Seaman, M.S., Velinzon, K., Pietzsch, J., Ott, R.G., Anthony, R.M., Zebroski, H., Hurley, A., et al. (2009). Broad diversity of neutralizing antibodies isolated from memory B cells in HIV-infected individuals. Nature *458*, 636–640.

Schmidt, A.G., Therkelsen, M.D., Stewart, S., Kepler, T.B., Liao, H.X., Moody, M.A., Haynes, B.F., and Harrison, S.C. (2015). Viral receptor-binding site antibodies with diverse germline origins. Cell *161*, 1026–1034.

Schroeder, K.M.S., Agazio, A., Strauch, P.J., Jones, S.T., Thompson, S.B., Harper, M.S., Pelanda, R., Santiago, M.L., and Torres, R.M. (2017). Breaching peripheral tolerance promotes the production of HIV-1-neutralizing antibodies. J. Exp. Med. *214*, 2283–2302.

Shlomchik, M.J., Aucoin, A.H., Pisetsky, D.S., and Weigert, M.G. (1987). Structure and function of anti-DNA autoantibodies derived from a single autoimmune mouse. Proc. Natl. Acad. Sci. USA *84*, 9150–9154.

Smith, K., Garman, L., Wrammert, J., Zheng, N.Y., Capra, J.D., Ahmed, R., and Wilson, P.C. (2009). Rapid generation of fully human monoclonal antibodies specific to a vaccinating antigen. Nat. Protoc. *4*, 372–384.

Smith, M.J., Packard, T.A., O'Neill, S.K., Henry Dunand, C.J., Huang, M., Fitzgerald-Miller, L., Stowell, D., Hinman, R.M., Wilson, P.C., Gottlieb, P.A., and Cambier, J.C. (2015). Loss of anergic B cells in prediabetic and new-onset type 1 diabetic patients. Diabetes *64*, 1703–1712.

Stein, A., and Kortemme, T. (2013). Improvements to robotics-inspired conformational sampling in rosetta. PLoS ONE *8*, e63090.

Sui, J., Hwang, W.C., Perez, S., Wei, G., Aird, D., Chen, L.M., Santelli, E., Stec, B., Cadwell, G., Ali, M., et al. (2009). Structural and functional bases for broad-spectrum neutralization of avian and human influenza A viruses. Nat. Struct. Mol. Biol. *16*, 265–273.

Tan, H.X., Jegaskanda, S., Juno, J.A., Esterbauer, R., Wong, J., Kelly, H.G., Liu, Y., Tilmanis, D., Hurt, A.C., Yewdell, J.W., et al. (2019). Subdominance and poor intrinsic immunogenicity limit humoral immunity targeting influenza HA stem. J. Clin. Invest. *129*, 850–862.

Thomson, C.A., Wang, Y., Jackson, L.M., Olson, M., Wang, W., Liavonchanka, A., Keleta, L., Silva, V., Diederich, S., Jones, R.B., et al. (2012). Pandemic H1N1 Influenza Infection and Vaccination in Humans Induces Cross-Protective Antibodies that Target the Hemagglutinin Stem. Front. Immunol. *3*, 87.

Tiller, T., Tsuiji, M., Yurasov, S., Velinzon, K., Nussenzweig, M.C., and Wardemann, H. (2007). Autoreactivity in human IgG+ memory B cells. Immunity *26*, 205–213.

Verkoczy, L., Diaz, M., Holl, T.M., Ouyang, Y.B., Bouton-Verville, H., Alam, S.M., Liao, H.X., Kelsoe, G., and Haynes, B.F. (2010). Autoreactivity in an HIV-1 broadly reactive neutralizing antibody variable region heavy chain induces immunologic tolerance. Proc. Natl. Acad. Sci. USA *107*, 181–186.

Volpe, J.M., and Kepler, T.B. (2009). Genetic correlates of autoreactivity and autoreactive potential in human Ig heavy chains. Immunome Res. *5*, 1.

Wardemann, H., Yurasov, S., Schaefer, A., Young, J.W., Meffre, E., and Nussenzweig, M.C. (2003). Predominant autoantibody production by early human B cell precursors. Science *301*, 1374–1377.

Weitzner, B.D., Kuroda, D., Marze, N., Xu, J., and Gray, J.J. (2014). Blind prediction performance of RosettaAntibody 3.0: grafting, relaxation, kinematic loop modeling, and full CDR optimization. Proteins *82*, 1611–1623.

Weitzner, B.D., Jeliazkov, J.R., Lyskov, S., Marze, N., Kuroda, D., Frick, R., Adolf-Bryfogle, J., Biswas, N., Dunbrack, R.L., Jr., and Gray, J.J. (2017). Modeling and docking of antibody structures with Rosetta. Nat. Protoc. *12*, 401–416.

Whittle, J.R., Zhang, R., Khurana, S., King, L.R., Manischewitz, J., Golding, H., Dormitzer, P.R., Haynes, B.F., Walter, E.B., Moody, M.A., et al. (2011). Broadly neutralizing human antibody that recognizes the receptor-binding pocket of influenza virus hemagglutinin. Proc. Natl. Acad. Sci. USA *108*, 14216–14221.

Wrammert, J., Smith, K., Miller, J., Langley, W.A., Kokko, K., Larsen, C., Zheng, N.Y., Mays, I., Garman, L., Helms, C., et al. (2008). Rapid cloning of high-affinity human monoclonal antibodies against influenza virus. Nature *453*, 667–671.

Wrammert, J., Koutsonanos, D., Li, G.M., Edupuganti, S., Sui, J., Morrissey, M., McCausland, M., Skountzou, I., Hornig, M., Lipkin, W.I., et al. (2011). Broadly cross-reactive antibodies dominate the human B cell response against 2009 pandemic H1N1 influenza virus infection. J. Exp. Med. *208*, 181–193.

Wu, N.C., Yamayoshi, S., Ito, M., Uraki, R., Kawaoka, Y., and Wilson, I.A. (2018). Recurring and Adaptable Binding Motifs in Broadly Neutralizing Antibodies to Influenza Virus Are Encoded on the D3-9 Segment of the Ig Gene. Cell Host Microbe *24*, 569–578.e4, e4.

Zarnitsyna, V.I., Ellebedy, A.H., Davis, C., Jacob, J., Ahmed, R., and Antia, R. (2015). Masking of antigenic epitopes by antibodies shapes the humoral immune response to influenza. Philos. Trans. R. Soc. Lond. B Biol. Sci. *370*, 370.

Zhang, J., and Yeh, M. (1994). Cloning, sequencing and analyzing of the heavy chain V region genes of human polyreactive antibodies. Cell Res. *4*, 31–46.

Zhang, J., Jacobi, A.M., Wang, T., Berlin, R., Volpe, B.T., and Diamond, B. (2009). Polyreactive autoantibodies in systemic lupus erythematosus have pathogenic potential. J. Autoimmun. *33*, 270–274.

Zhang, R., Verkoczy, L., Wiehe, K., Munir Alam, S., Nicely, N.I., Santra, S., Bradley, T., Pemble, C.W.T., 4th, Zhang, J., Gao, F., et al. (2016). Initiation of immune tolerance-controlled HIV gp41 neutralizing B cell lineages. Sci. Transl. Med. *8*, 336ra62.

STAR★METHODS

KEY RESOURCES TABLE

REAGENT or RESOURCE	SOURCE	IDENTIFIER
**Antibodies**		
Anti-human CD19 PE-AF610 conjugate	Invitrogen/Thermo Scientific	Cat# MHCD1922 RRID: AB_10373379
Anti-human CD27 R-PE conjugate	Invitrogen/Thermo Scientific	Cat# MHC2704 RRID: AB_10392393
Anti-human CD38 APC-Cy5.5 conjugate	Invitrogen/Thermo Scientific	Cat# MHCD3819 RRID: AB_10371760
Anti-human CD3 FITC conjugate	Invitrogen/Thermo Scientific	Cat# MHCD0301 RRID: AB_10376003
Anti-human CD20 FITC conjugate	Invitrogen/Thermo Scientific	Cat# MHCD2001 RRID: AB_10373690
RosetteSep human B cell enrichment cocktail	StemCell Technologies	Cat#15064
HRP-conjugated goat anti-human IgG antibody	Jackson Immuno Research	Cat# 109-035-098 RRID: AB_2337586
Streptavidin-HRP	Southern Biotech	Cat#7100-05
**Bacterial and Virus Strains**		
NEB® 5-alpha Competent *E. coli*	NEB	Cat# C2988J
A/Solomon Islands/6/2006 (H1N1)	Patrick Wilson's laboratory stock	N/A
A/Brisbane/59/2007(H1N1)	Patrick Wilson's laboratory stock	N/A
A/California/7/2009 (H1N1)	Patrick Wilson's laboratory stock	N/A
A/New Caledonia/20/1999 (H1N1)	Patrick Wilson's laboratory stock	N/A
A/Brazil/11/1978 (H1N1)	Patrick Wilson's laboratory stock	N/A
A/Chile/1/1983 (H1N1)	Patrick Wilson's laboratory stock	N/A
A/Texas/36/1991 (H1N1)	Patrick Wilson's laboratory stock	N/A
A/Michigan/45/2015 (H1N1)	Patrick Wilson's laboratory stock	N/A
A/Puerto Rico/8/1934 (H1N1)	Patrick Wilson's laboratory stock	N/A
A/swine/Mexico/AVX8/2011 (H1N2)	Patrick Wilson's laboratory stock	N/A
A/Wisconsin/57/2005 (H3N2)	Patrick Wilson's laboratory stock	N/A
A/Uruguay/716/2007 (H3N2)	Patrick Wilson's laboratory stock	N/A
A/Texas/50/2012 (H3N2)	Patrick Wilson's laboratory stock	N/A
A/Switzerland/9715293/2013 (H3N2)	Patrick Wilson's laboratory stock	N/A
A/Hong Kong/4801/2014 (H3N2)	Patrick Wilson's laboratory stock	N/A
B/Phuket/3073/2013	Patrick Wilson's laboratory stock	N/A
B/Brisbane/60/2008	Patrick Wilson's laboratory stock	N/A
**Chemicals, Peptides, and Recombinant Proteins**		
A/California/7/2009 (H1N1) HA	Florian Krammer's laboratory stock	N/A
A/California/4/2009 (H1N1) NA	Florian Krammer's laboratory stock	N/A
A/South Carolina/1/1918 (H1N1) HA	Florian Krammer's laboratory stock	N/A
B/Brisbane/60/2008 HA	Florian Krammer's laboratory stock	N/A
A/Vietnam/1203/2004 (H5N1) HA	Florian Krammer's laboratory stock	N/A
Chimeric H6/1 HA (H6 head from A/mallard/Sweden/81/2002 combined with H1 stalk from A/California/04/2009)	Florian Krammer's laboratory stock	N/A
Chimeric H5/1 HA (H5 head from A/mallard/Sweden/24/2002 combined with H1 stalk from A/California/4/2009)	Florian Krammer's laboratory stock	N/A
Mini-HA (H1 stalk domain from A/Brisbane/59/2007)	Lynda Coughlan's laboratory stock	N/A
B/Massachusetts/02/2012 HA	Protein Sciences Corp.	Lot #1208-106

(*Continued on next page*)

Continued

REAGENT or RESOURCE	SOURCE	IDENTIFIER
A/Anhui/1/2013 (H7N9) HA	BEI Resources	Cat#NR44365
Cardiolipin solution from bovine heart	Sigma-Aldrich	Cat#SRE0029
Calf thymus DNA	Thermo Scientific	Cat#15633019
Ultrapure flagellin from *Salmonella typhimirium*	Invivogen	Cat#tlrl-epstfla-5
Recombinant Human Insulin	Sigma-Aldrich	Cat#I2643
LPS from *Eschericia coli* O55:B5	Sigma-Aldrich	Cat#L2880
Keyhole Limpet Hemocyanin	Millipore	Cat#374825
PEI 25K, Transfection Grade	Polysciences	Cat# 23966-2
Super Aquablue ELISA substrate	Thermo Scientific	Cat# 00-4203-58
EZ-link Sulfo-NHS-Biotin	Thermo Scientific	Cat# 21217
Trypsin, TPCK treated	Sigma-Aldrich	Cat# T8802
Pierce™ Protein A agarose	Thermo Scientific	Cat# 20334
Lymphocyte Separation Medium	Corning	Cat#25-072-CV
20x HBS-EP+ Running Buffer	VWR Teknova	Cat#76102-774
Amine coupling kit	Cytiva	Cat#BR100050
Acetate buffer	Cytiva	Cat#BR100351
CM5 sensor chip series S	Cytiva	Cat#BR100530
ProteOn Phosphoric Acid Solution (0.85%)	BioRad	Cat#1762260
Streptavidin Biosensors	FortéBio	Cat#185019
Protein A Biosensors	FortéBio	Cat#185010
Experimental Models: Cell Lines		
MDCK cells	ATCC	Cat# CCL-34
HEK293T Cell Line	ATCC	Cat# CRL-11268
Experimental Models: Organisms/Strains		
Specific Pathogen Free Fertilized Eggs	Charles River	Cat#10100326
Recombinant DNA		
IgG-AbVec	Patrick Wilson's laboratory stock	N/A
Igκ-AbVec	Patrick Wilson's laboratory stock	N/A
Igλ-AbVec	Patrick Wilson's laboratory stock	N/A
Software and Algorithms		
GraphPad Prism (version 8.4.3)	GraphPad Software Inc	http://www.graphpad.com; RRID: SCR_002798
Jmp (version 15.0)	SAS	https://www.jmp.com/en_us/software.html; RRID: SCR_014242
IMGT/V-QUEST	Immunogenetics, Marie-Paule Lefranc	http://www.imgt.org/IMGT_vquest/share/textes/; RRID: SCR_010749
IgBlast	NCBI	http://www.ncbi.nlm.nih.gov/igblast/; RRID: SCR_002873
Clustal Omega	EMBL-EBI	http://www.ebi.ac.uk/Tools/msa/clustalo/; RRID: SCR_001591
UCSF Chimera	Resource for Biocomputing Visualization and Informatics	https://www.cgl.ucsf.edu/chimera/; RRID: SCR_004097
FortéBio Data Analysis Software (version 9)	FortéBio	Cat#50-0293
Biacore Insight Evaluation Software (version 1)	Cytiva	Cat#29310602
Rosetta Antibody Modeler	University of Washington	https://www.rosettacommons.org/; RRID: SCR_015701
MOE	Chemical Computing Group	https://www.chemcomp.com/; RRID SCR_014882

(*Continued on next page*)

CellPress
OPEN ACCESS

Continued

REAGENT or RESOURCE	SOURCE	IDENTIFIER
AMBER Simulation Software	AmberMD	https://ambermd.org/; RRID: SCR_014230
Ambertools19	AmberMD	https://ambermd.org/; RRID: SCR_018497
Pymol	Schrödinger	https://pymol.org/2/; RRID: SCR_000305

RESOURCE AVAILABILITY

Lead Contact

Further information and requests for resources and reagents should be directed to the Lead Contact, Patrick C. Wilson (wilsonp@uchicago.edu).

Materials Availability

There are restrictions to the availability of mAbs from this study due to the lack of an external centralized repository for its distribution and our need to maintain the stock. We are glad to share mAbs with reasonable compensation by requestor for its processing and shipping.

Data and Code Availability

This study did not generate any unique datasets or code.

EXPERIMENTAL MODEL AND SUBJECT DETAILS

Human Materials

Human PBMCs were obtained from multiple subjects from multiple cohorts, which is outlined in Table S1 and Table S2. All studies were performed with the approval of the University of Chicago Institutional Review Board (ID #09-043-A). The H7N9 vaccine study cohort is identified as clinical trial NCT01995695 and the chimeric HA vaccine study cohort is identified as clinical trial NCT03300050.

Cell Lines

Human Embryonic Kidney HEK293T (female, # CRL-11268) and Madin Darby Canine Kidney MDCK (female, # CCL-34, NBL-2) cells were purchased and authenticated by the American Type Culture Collection (ATCC). All cells were maintained in a humidified atmosphere of 5% CO_2 at 37°C. HEK293T cells were maintained in Advanced-DMEM supplemented with 2% ultra-low IgG Fetal Bovine Serum (FBS) (Invitrogen), 1% L-Glutamine (Invitrogen) and 1% antibiotic-antimycotic (Invitrogen). MDCK cells were maintained in DMEM supplemented with 10% FBS (Invitrogen), 1% L-Glutamine (Invitrogen) and 1% Penicillin-Streptomycin (Invitrogen).

METHOD DETAILS

Monoclonal antibody production

Monoclonal antibodies were generated as previously described (Guthmiller et al., 2019; Wrammert et al., 2008; Smith et al., 2009). Peripheral blood was obtained from each subject approximately 7 days after vaccination or infection, or bait-sorted B cells were obtained 28+ days post-vaccination. Lymphocytes were isolated and enriched for B cells using RosetteSep. Plasmablasts ($CD3^-CD19^+CD27^{hi}CD38^{hi}$) were single-cell sorted into 96-well plates. Immunoglobulin heavy and light chain genes were amplified by reverse transcriptase polymerase chain reaction (RT-PCR), sequenced, cloned into human IgG1, human kappa chain, or human lambda expression vectors, and co-transfected into human embryonic kidney (HEK) 293T cells. Secreted mAbs were purified from the supernatant using protein A agarose beads. For germline mAbs against the stalk domain, cH5/1-binding B cells were sorted from subjects 28 days after cH5/1 vaccination (NCT03300050). mAb heavy chain and light chain sequences were synthesized from single-cell RNA-sequencing data of cHA-baited B cells (IDT), and cloned into the human IgG1, human kappa chain, or human lambda expression vectors. For reverted germline mAbs, germline sequences were determined using IgBlast and synthesized (IDT), and cloned into the human IgG1, human kappa chain, or human lambda expression vectors. Previously published mAb sequences were obtained from GenBank, synthesized (IDT), and cloned into the human IgG1, human kappa chain, or human lambda expression vectors. B cell clones were determined by aligning all the V(D)J sequences sharing identical progenitor sequences, as predicted by IgBLAST using our in-house software, Vgenes.

Viruses and recombinant proteins

Influenza viruses used in all assays were grown in-house in specific pathogen free (SPF) eggs, harvested, purified, and titered. The A/swine/Mexico/AVX8/2011 H1N2 virus (Mena et al., 2016) was provided by Ignacio Mena, Adolfo García-Sastre, and Sean Liu

at Icahn School of Medicine at Mount Sinai. Recombinant HA, NA, and NP derived from A/California/7/2009 (H1N1), A/South Carolina/1/1918, A/Hong Kong/4801/2014 (H3N2), A/Switzerland/9715293/2013 (H3N2), A/Texas/50/2012 (H3N2), A/Vietnam/1203/2004 (H5N1), and A/Anhui/1/2013 (H7N9) were obtained from BEI resources or kindly provided from the Krammer laboratory at Icahn School of Medicine at Mount Sinai and the Hensley laboratory at The University of Pennsylvania. Chimeric HA and Mini-HA headless stalk proteins were kindly provided by Florian Krammer and Lynda Coughlan, respectively, at Icahn School of Medicine at Mount Sinai.

Antigen-Specific ELISA

High protein-binding microtiter plates (Costar) were coated with 8 hemagglutination units (HAU) of virus in carbonate buffer or with recombinant HA, NA, or NP, or mini-HA (Impagliazzo et al., 2015) at 1 μg/mL in phosphate-buffered saline (PBS) overnight at 4°C. Plates were washed the next morning with PBS 0.05% Tween and blocked with PBS containing 20% fetal bovine serum (FBS) for 1 h at 37°C. Antibodies were then serially diluted 1:3 starting at 10 μg/mL and incubated for 1.5 h at 37°C. Horseradish peroxidase (HRP)-conjugated goat anti-human IgG antibody diluted 1:1000 (Jackson Immuno Research) was used to detect binding of mAbs, and plates were subsequently developed with Super Aquablue ELISA substrate (eBiosciences). Absorbance was measured at 405 nm on a microplate spectrophotometer (BioRad). To standardize the assays, control antibodies with known binding characteristics were included on each plate, and the plates were developed when the absorbance of the control reached 3.0 OD units. MAbs represented as binding unknown influneza antigens bound whole virus but none of the individual antigens tested. Similarly, mAbs that did not definitively bind the HA head or stalk are listed as binding unknown HA^{+} epitopes. To determine which mAbs bound the conserved HA stalk region, competition ELISAs were carried out using the known stalk-binding mAb CR9114 as a competitor mAb (Dreyfus et al., 2012), or by performing ELISAs using a chimeric HA (cH5/1) and mini-HA protein. Competition ELISAs were performed by inhibiting binding of a biotinylated antibody of interest at the half-maximal binding concentration with a 10+ fold molar excess of competitor antibody. HRP-conjugated streptavidin diluted 1:1000 (Southern Biotech) was used for detection. Plates were developed until samples in the absence of competitor antibody reached an OD of 1.0 (Henry Dunand et al., 2015). All experiments were performed in duplicate and replicated 2-3 times.

Polyreactive ELISA

Polyreactive ELISAs were performed as previously described (Bunker et al., 2017; Andrews et al., 2015a; Koelsch et al., 2007). High-protein binding microtiter plates (Costar) were coated with 10 μg/mL calf thymus dsDNA (Thermo Fisher Scientific), 2 μg/mL *Salmonella enterica* serovar Typhimurium flagellin (Invitrogen), 5 μg/mL human insulin (Sigma-Aldrich), 10 μg/mL KLH (Invitrogen), and 10 μg/mL *Escherichia coli* LPS (Sigma-Aldrich) in PBS. Plates were coated with 10 μg/mL cardiolipin in 100% ethanol and allowed to dry overnight. Plates were washed with water and blocked with PBS/0.05%Tween/1mM EDTA. MAbs were diluted 1 μg/mL in PBS and serially diluted 4-fold, and added to plates for 1.5 h. Goat anti-human IgG-HRP (Jackson Immunoresearch) was diluted 1:2000 in PBS/0.05%Tween/1mM EDTA. Plates were developed with Super Aquablue ELISA substrate (eBioscience) until the positive control mAb, 3H9 (Shlomchik et al., 1987), reached an A_{450} of 3. All experiments were performed in duplicate and replicated 2-3 times.

Microneutralization and Hemagglutination Inhibition Assays (HAI)

Madin-Darby canine kidney cells (MDCK; ATCC) were maintained in culture at 37°C with 5% CO_2 in Dulbecco's Modified Eagle Medium (DMEM, GIBCO) supplemented with 10% fetal bovine serum (FBS, GIBCO), 2 mM L-glutamine (GIBCO), and penicillin and streptomycin (GIBCO). Microneutralization assay for mAb characterization was carried out as previously described (Henry Dunand et al., 2015; Chen et al., 2018). MDCK cells were maintained in DMEM supplemented with 10% FBS, 1% penicillin-streptomycin, and 1% L-glutamine at 37°C with 5% CO_2. The day before the experiment, 25,000 MDCK cells were added to each well of a 96-well plate. Serial two-fold dilutions of mAb were mixed with an equal volume of 100 50% tissue culture infectious doses ($TCID_{50}$) of virus for 1 h and added to MDCK cells for 1 h at 37°C. The mixture was removed, and cells were cultured for 20 h at 37°C with 1X MEM supplemented with 1 μg/mL tosyl phenylalanyl chloromethyl ketone (TPCK)-treated trypsin and appropriate mAb concentration. Cells were washed twice with PBS, fixed with 80% ice cold acetone at 20°C for at least 1 h, washed 3 times with PBS, blocked for 30 min with 3% BSA, and then treated for 30 min with 2% H_2O_2. Cells were incubated with a mouse anti-NP antibody (1:1000; Millipore) in 3% BSA-PBS for 1 h at room temperature (RT), followed by goat anti-mouse IgG HRP (1:1000; Southern Biotech) in 3% BSA-PBS for 1 h at RT. The plates were developed with Super Aquablue ELISA substrate at 405 nm until virus only controls reached an OD of 1. The signal from uninfected wells was averaged to represent 100% inhibition. The signal from infected wells without mAb was averaged to represent 0% inhibition. Duplication wells were used to calculate the mean and SD of neutralization, and inhibitory concentration 50 (IC_{50}) was determined by a sigmoidal dose response curve. The inhibition ratio (%) was calculated as below: ((OD Pos. Control – OD Sample) / (OD Pos. Control – OD Neg. Control)) * 100. The final IC_{50} was determined using Prism software (GraphPad).

For HAI assays, viruses were diluted to 8 HAU/50 μl. 25 μL was combined with an equal volume of mAb (starting concentration 30 μg/mL), serially diluted 1:2 in PBS in duplicate, and subsequently incubated at RT for 1 h. 50 μL of 0.5% Turkey red blood cells (Lampire Biological) were added to each well and incubated for 45 min at RT. Minimum effective concentrations were then calculated based on the final dilution of mAb for which hemagglutination inhibition was observed. All experiments were performed in duplicate twice.

CellPress
OPEN ACCESS

Molecular Dynamics Simulations

To predict the structure of the variable fragment (Fv) of the CDR-sequences, we applied the program RosettaAntibody (Weitzner et al., 2017; Weitzner et al., 2014; Stein and Kortemme, 2013). We assume that the structural modeling works reliably for five of the six CDR loop regions, i.e., those that can be characterized by canonical structures (Chothia and Lesk, 1987; Chothia et al., 1989). MD simulations were performed with the AMBER18 simulation package. All structures were prepared in MOE (Molecular Operating Environment, Chemical Computing Group, version 2018.0901) using the protonate3D tool (Labute, 2009). With tleap of the AmberTools18 package, all three systems were soaked into a cubic water boxes of TIP3P water molecules with a minimum wall distance of 10 Å (Jorgensen et al., 1983). Parameters for all antibody models derive from the AMBER force field 14SB (Maier et al., 2015). All antibody Fvs were carefully equilibrated using a multistep equilibration protocol. MD simulations as well as all accelerated MD (aMD) simulations were performed in an NpT ensemble using pmemd.cuda (Salomon-Ferrer et al., 2013). Bonds involving hydrogen atoms were restrained by applying the SHAKE (Miyamoto and Kollman, 1992) algorithm, allowing a time step of 2.0 fs. Atmospheric pressure of the system was preserved by weak coupling to an external bath using the Berendsen algorithm (Berendsen et al., 1984). The Langevin thermostat (Adelman and Doll, 1975) was used to maintain the temperature at 300 K during simulations. All aMD (Hamelberg et al., 2004) simulations were performed using the dual-boost protocol implemented in pmemd.cuda (Salomon-Ferrer et al., 2013). Thereby, the total potential is accelerated, and an extra boosting is applied to the dihedral potential (Hamelberg et al., 2007). All simulations were analyzed using cpptraj (Roe and Cheatham, 2013) in AmberTools18, the reweighting protocol provided by Miao et al., and in-house python (Millman and Aivazis, 2011) scripts. The free energy profile of the backbone torsions of the HC-CDR3 loop and the whole Fv were reconstructed from the aMD simulations via Boltzmann reweighting using a Maclaurin series expansion (up to the tenth order) as the approximation for the exponential term (Miao et al., 2014).

HA models

The structure of A/California/7/2009 HA (PDB 4M4Y) was downloaded from the Protein Database, and the footprints of three mAbs binding conserved epitopes are shown: CH65 (RBS; PDB 5UGY), Fab6649 (Lateral Patch; PDB 5W6G), and CR9114 (BN Stalk Epitope; PDB 4FQI). Structure alignment and figures were made using UCSF Chimera (Pettersen et al., 2004) and Adobe Photoshop. Individual monomer of the HA trimer is indicated in gray.

Size Exclusion Chromatography

Protein concentrations were determined by A280 using a calculated extinction coefficient. Large aggregates were removed by 0.22um spin filter prior to injection on size exclusion chromatography. Purified mAbs were loaded onto pre-conditioned column (Superdex 200 10/300 GL, GE Healthcare) with PBS at a flow rate of 0.5 mL/min and room temperature. At tested concentrations only single peaks were observed eluting from the column corresponding to approximate size of mAb monomer.

Biolayer Interferometry (BLI)

mAb binding competition was performed using biolayer interferometer (ForteBio). For competition of HA-KLH, 10 μg/mL of polyreactive mAbs were loaded onto a Protein A probe for 300 s, followed by incubation with 10 μg/mL A/California/7/2009 HA for 420 s, and incubation with 20 μg/mL or 100 μg/mL of KLH for 720 s. For KLH-HA competition, 10 μg/mL of polyreactive mAbs were loaded onto a Protein A probe for 300 s, followed by incubation with 20 μg/mL or 100 μg/mL of KLH for 720 s, and incubation with 10 μg/mL A/California/7/2009 HA for 420 s. For K_d measurements, biotinylated A/California/7/2009 HA (10 μg/mL) was loaded onto streptavidin probes for 150 s, followed by association with mAbs (1.25-20 μg/mL) for 480 s, and dissociation for 480 s. K_d were determined using ForteBio analysis software.

Surface Plasmon Resonance (SPR)

All SPR was performed on a GE Biacore 8K SPR instrument within the Biophysics core at the University of Chicago. MAbs were diluted to 5 μg/mL in acetate buffer and were immobilized to CM5 sensor chips (Cytiva) using the amine coupling kit (Cytiva). A/California/7/2009 HA was diluted to 32 nM and diluted two-fold (2-32 nM) and loaded into a 96-well plate. ProteinOn solution (BioRad) was included in-between HA dilutions to regenerate chip between HA concentrations. HA was run over the chip for 300 s (30 μl/min) and was followed by a 600 s dissociation step. ProteinOn solution was run across the chip for 60 s (100 μl/min). K_a and K_d calculations were determined by using the Biacore analysis software.

QUANTIFICATION AND STATISTICAL ANALYSIS

All statistical analysis was performed using Prism software (Graphpad Version 7.0) or *R*. Chi-square tests across datasets were corrected for multiple comparisons using post hoc Chi-square test. Sample sizes (n) for the number of mAbs tested are indicated in corresponding figures or in the center of pie graphs. Number of biological repeats for experiments and specific tests for statistical significance used are indicated in the corresponding figure legends. *P value*s less than or equal to 0.05 were considered significant. * $P \leq 0.05$, ** $P \leq 0.01$, *** $P \leq 0.001$, **** $p < 0.0001$.

Immunity, Volume *53*

Supplemental Information

Polyreactive Broadly Neutralizing B cells Are Selected to Provide Defense against Pandemic Threat Influenza Viruses

Jenna J. Guthmiller, Linda Yu-Ling Lan, Monica L. Fernández-Quintero, Julianna Han, Henry A. Utset, Dalia J. Bitar, Natalie J. Hamel, Olivia Stovicek, Lei Li, Micah Tepora, Carole Henry, Karlynn E. Neu, Haley L. Dugan, Marta T. Borowska, Yao-Qing Chen, Sean T.H. Liu, Christopher T. Stamper, Nai-Ying Zheng, Min Huang, Anna-Karin E. Palm, Adolfo García-Sastre, Raffael Nachbagauer, Peter Palese, Lynda Coughlan, Florian Krammer, Andrew B. Ward, Klaus R. Liedl, and Patrick C. Wilson

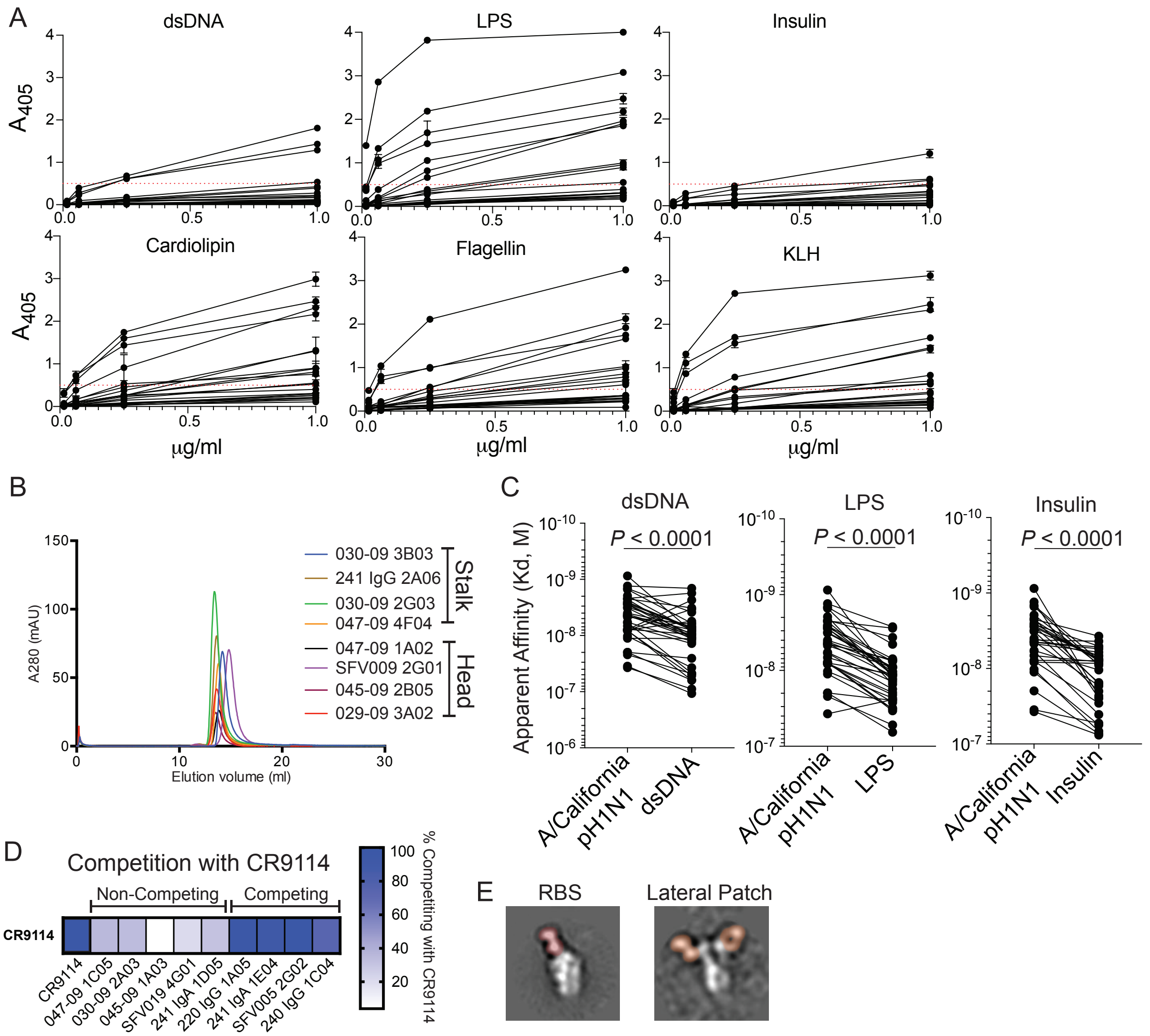

Figure S1: Polyreactive binding of influenza virus-binding antibodies. Related to Figure 1. (**A**) Polyreactivity ELISA example graphs. Representative of 22 antibodies tested for polyreactivity against the 6 antigens used in the polyreactivity ELISA panel. (**B**) Size exclusion chromatography of polyreactive mAbs indicating that polyreactive mAbs are monomers and do not form aggregates. (**C**) Paired apparent affinity (K_d) of polyreactive mAbs binding to A/California/7/2009 (pH1N1) virus and dsDNA (n=37), insulin (n=30), or LPS (n=36). Each line connects the same mAb. (**D**) Representative competition ELISA results of mAbs competing or not competing with CR9114, an antibody that specifically targets the BN stalk epitope. (**E**) Representative negative stain electron microscopy of an RBS binding antibody (SFV018 2D01 fab in red) and lateral patch binding antibody (045-09 2B05 fab in orange). Data in **C** were analyzed by paired non-parametric Wilcoxon matched-pairs signed rank Tests. Limit of Detection (L.O.D.) represented as dashed red line.

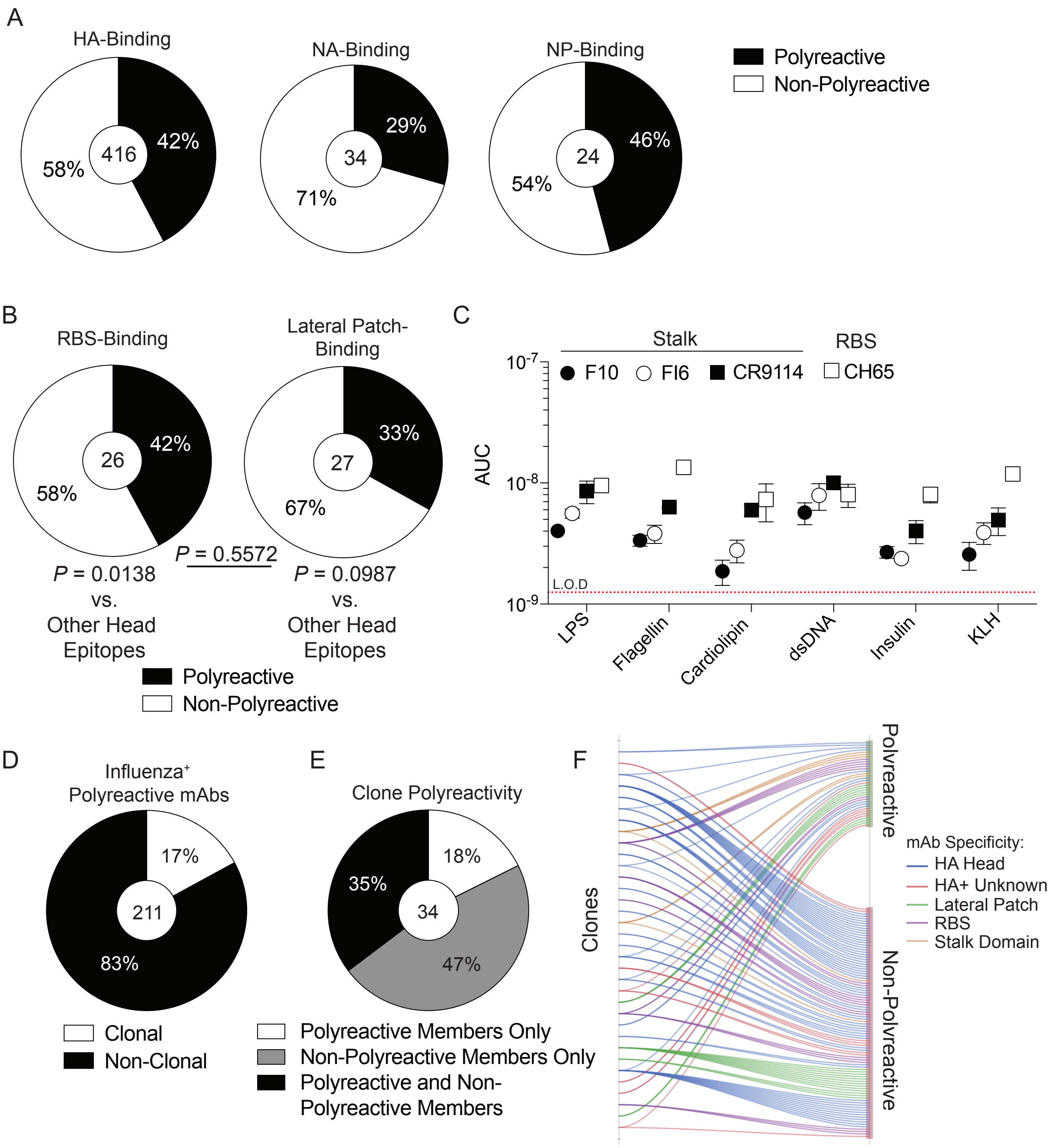

Figure S2: Polyreactivity of antigen-specific mAbs. Related to Figure 1. (**A**) Proportion of mAbs binding HA, NA, and NP that are polyreactive. (**B**) Proportion of RBS or lateral patch-binding mAbs that are polyreactive. (**C**) Polyreactivity of 4 published broadly neutralizing mAbs. The number in the center of pie graphs indicates the number of mAbs tested. Each antibody was tested in duplicate twice and the data are mean ± S.E.M. (**D**) Proportion of influenza virus positive polyreactive mAbs that are part of a clonal expansion. (**E**) Proportion of clones that only have polyreactive members, non-polyreactive members, or a mix of polyreactive and non-polyreactive

members. (**F**) Polyreactivity of clones based on antigen specificity. Each clone line on the left-hand side is one clonal expansion against HA (n=34 clones). For data in **A**, **B**, and **D**, the number in the center of each pie-graph is the number of mAbs tested. For data in **E**, the number in the center of the pie graph is the number of influenza virus specific clones analyzed. Data in **B** were analyzed by Fisher's Exact Test relative to other head epitope data in **Figure 1D**. Limit of Detection (L.O.D.) represented as dashed red line.

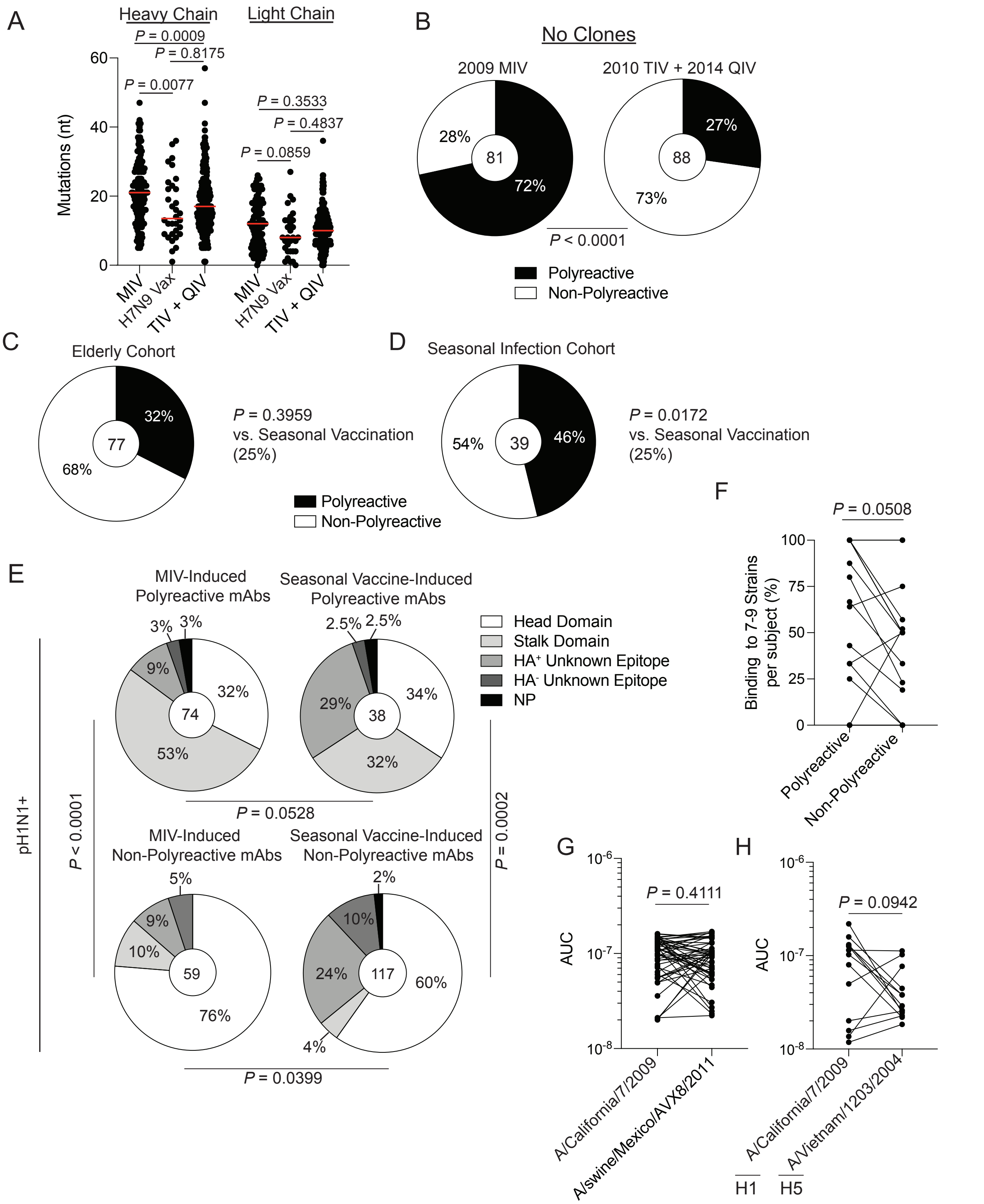

Figure S3: Polyreactive mAb induction by different influenza exposures and cross-reactivity of polyreactive mAbs. Related to Figure 2 and Figure 3. (**A**) Number of nucleotide mutations of heavy and light chains of mAbs generated from the 2009 MIV (heavy n=131; light n=123), H7N9 vaccine (heavy n=32; light n=31), and seasonal vaccination (TIV+QIV; heavy

n=259; light n=249). (**B**) Proportion of pH1N1+ mAbs that are polyreactive from individuals vaccinated with the 2009 MIV or 2010-2011 TIV + 2014-2015 QIV, excluding any clonal expansions. (**C**-**D**) MAbs isolated from elderly subjects (≥ 65 years old) immunized with seasonal influenza vaccines (**C**) or from adults infected with seasonal influenza A viruses (**D**) were tested for polyreactivity. (**E**) Epitope targeting of polyreactive and non-polyreactive mAbs induced by the 2009 MIV (left) or seasonal vaccination (right). (**F**) Proportion of polyreactive and non-polyreactive mAbs per subject (n=12) binding to 7-9 H1N1 strains, based on data in **Figure 3A**. Each line connects the proportion of polyreactive and non-polyreactive mAbs binding 7-9 H1N1 strains from each subject. (**G**) Binding affinity (as shown as AUC) of polyreactive mAbs (n=50) induced by the 2009 MIV against A/California/7/2009 and A/swine/Mexico/AVX8/2011 (H1N2). (**H**) Binding affinity (as shown as AUC) polyreactive mAbs (n=13) induced by the 2009 MIV and the 2014 QIV against A/California/7/2009 and A/Vietnam/1203/2004 recombinant H5. For data in **A**, each symbol represents one mAb and the red bar is the median. Lines in **F** and **G** connect the same mAb binding A/California/7/2009 and A/swine/Mexico/AVX8/2011 (**F**) or A/Vietnam/1203/2004 rH5 (**G**). For data in **B**-**E**, the number in the center of each pie graph is the number of mAbs tested. Data in **A** were analyzed by a non-parametric Kruskal-Wallis Test, data in **B**-**D** were analyzed by Fisher's Exact Test, data in **E** were analyzed by using Chi-square Tests, and data in **F**-**H** were analyzed by a paired non-parametric Wilcoxon matched-pairs signed rank Test.

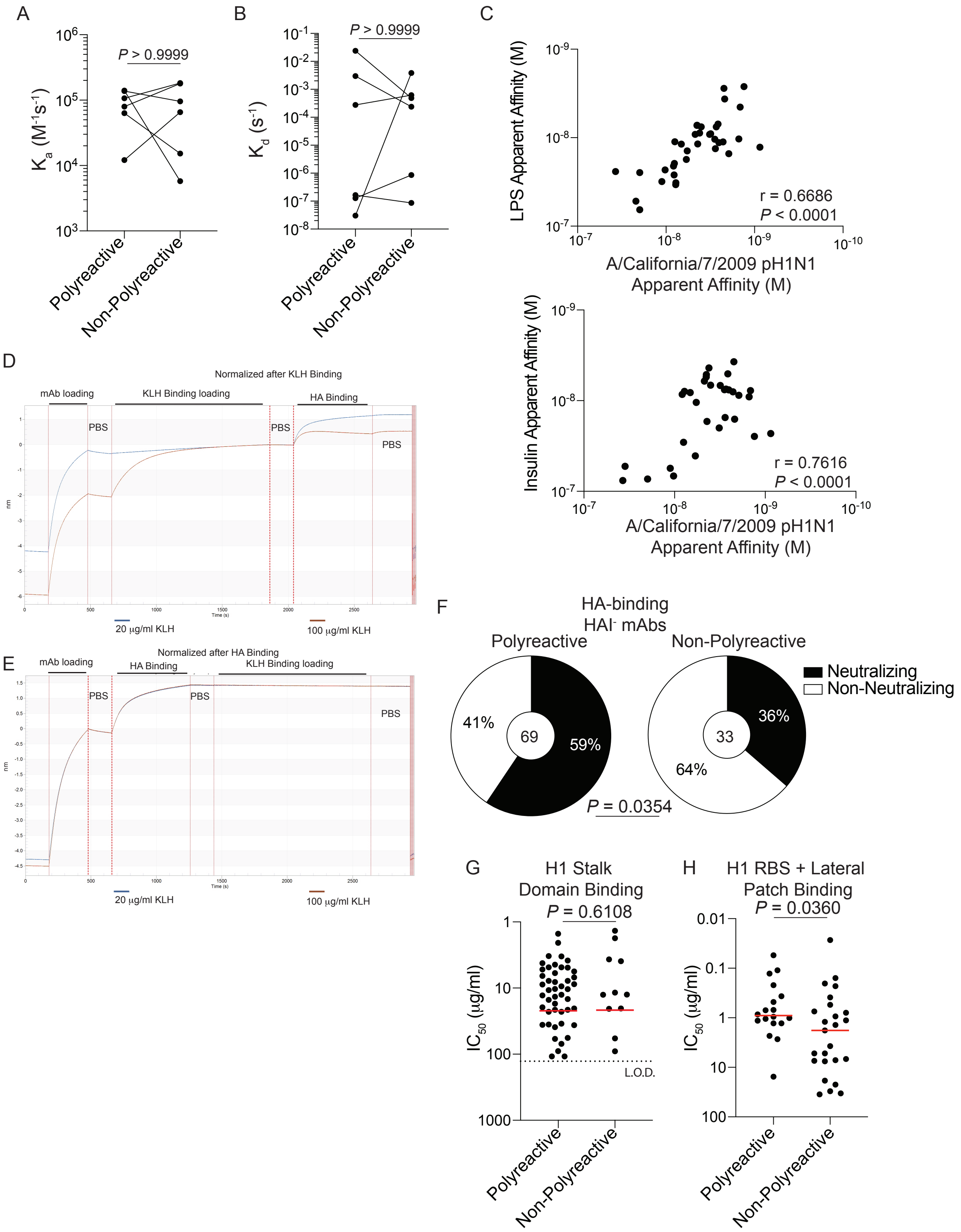

Figure S4: Polyreactivity augments viral binding and neutralization. Related to Figure 4. (**A-B**) K_a (**A**) and K_d (**B**) of polyreactive and non-polyreactive mAbs from the same clone binding to A/California/7/2009 HA. Each line connects polyreactive and non-polyreactive clonal members

(n=6). (**C**) Spearman Correlation of the apparent affinity (K_d) of polyreactive mAb binding to A/California/7/2009 virus and LPS (top; n=36) or Insulin (bottom; n=30). (**D**-**E**) Using biolayer interferometry, a Protein A sensor was loaded with SFV005 2G02 (polyreactive mAb). (**D**) The sensor was then dipped in 20 μg/ml or 100 μg/ml of KLH, followed by 10 μg/ml A/California/7/2009 HA. (**E**) After SFV005 2G02, the sensor was dipped into 10 μg/ml A/California/7/2009 HA, and then dipped in 20 μg/ml or 100 μg/ml of KLH. Data are representative of 10 mAbs. The assays were performed twice for each antibody. (**F**) Polyreactive and non-polyreactive antibodies targeting HA^+ HAI^- epitopes were tested for neutralization against A/California/7/2009. Proportion of polyreactive and non-polyreactive antibodies that are neutralizing. (**G**-**H**) Neutralization potency (IC_{50}) against A/California/7/2009 virus of polyreactive (n=47) and non-polyreactive (n=11) mAbs targeting the stalk domain (**G**) and of polyreactive (n=18) and non-polyreactive (n=25) mAbs targeting the RBS and lateral patch (**H**). For data in **C**, **G**, and **H**, each symbol represents one mAb and the red bar indicates the median. For data in **F**, the number in the center of each pie graph is the number of mAbs tested. Data in **A** and **B** were analyzed by a paired non-parametric Wilcoxon matched-pairs signed rank Test. Data in **F** were analyzed using a Fisher's Exact Test and data in **G** and **H** were analyzed using a non-parametric Mann-Whitney Test. Limit of Detection (L.O.D.) represented as dashed black line.

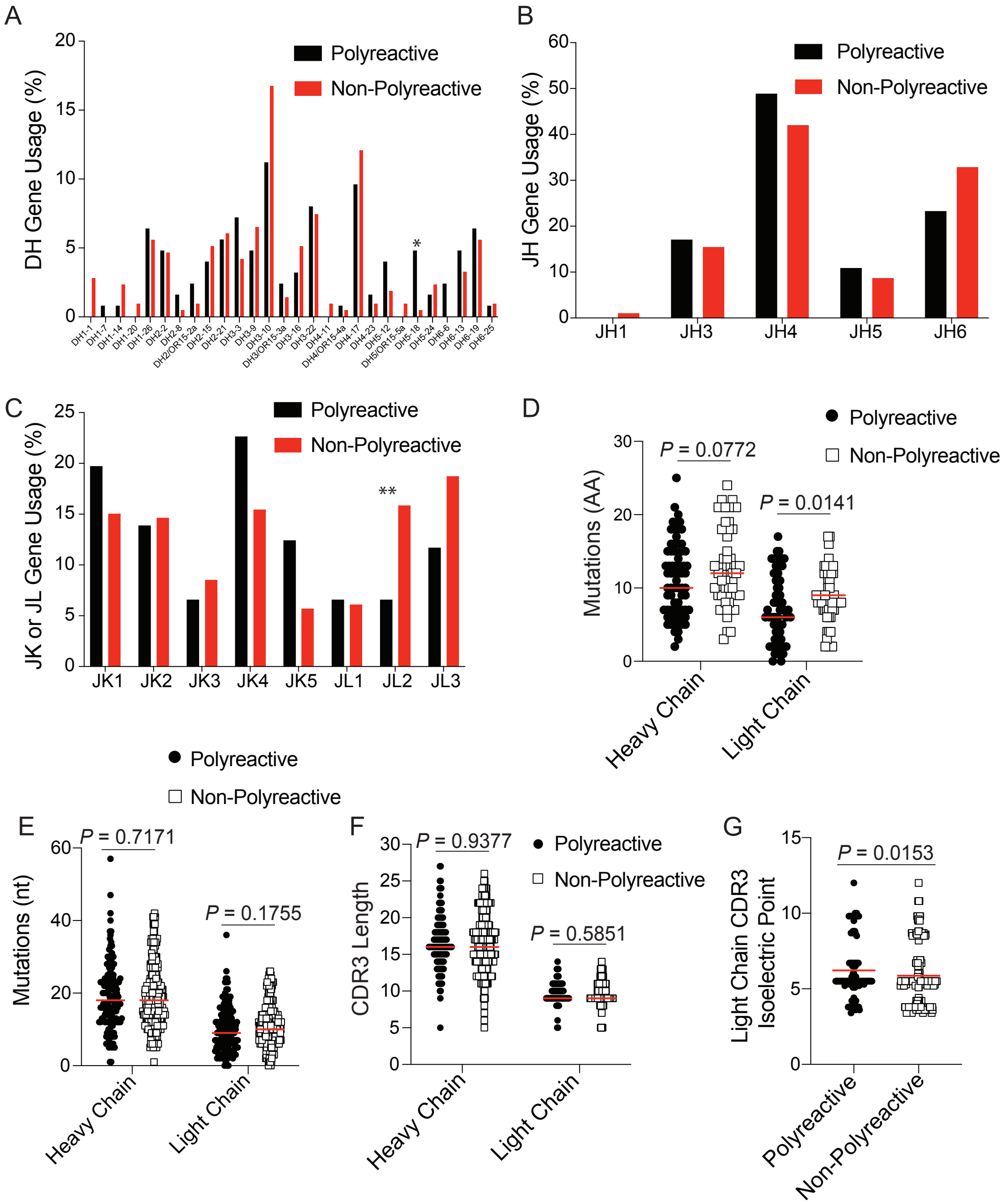

Figure S5: Repertoire and biochemical characteristics of polyreactive and non-polyreactive antibodies. Related to Figure 5. (**A-B**) DH (**A**) and JH (**B**) gene usage by polyreactive and non-polyreactive antibodies. (**C**) JK or JL gene usage by polyreactive and non-polyreactive antibodies. (**D**) Somatic hyper mutations (amino acid changes) of polyreactive (heavy n=71; light 68) and non-polyreactive (heavy n=55; light n=53) mAbs induced by the 2009 MIV. (**E**) Somatic hypermutations (nucleotide mutations) of heavy and light chains of all polyreactive (n=137) and non-polyreactive (n=246) mAbs. (**F**) Heavy chain and light chain CDR3 length of

polyreactive (n=137) and non-polyreactive mAbs (heavy n=245; light n=246). (**G**) Light chain CDR3 isoelectric point of polyreactive (n=137) and non-polyreactive (n=246) mAbs. For data in **D**-**G**, each symbol represents one mAb and the red bar indicates the median. Data in **A**-**C** were analyzed by Fisher's Exact Tests, and data in **D**-**G** were analyzed by unpaired non-parametric Mann-Whitney Tests. Each symbol represents a single antibody. * $P \leq 0.05$; ** $P \leq 0.01$

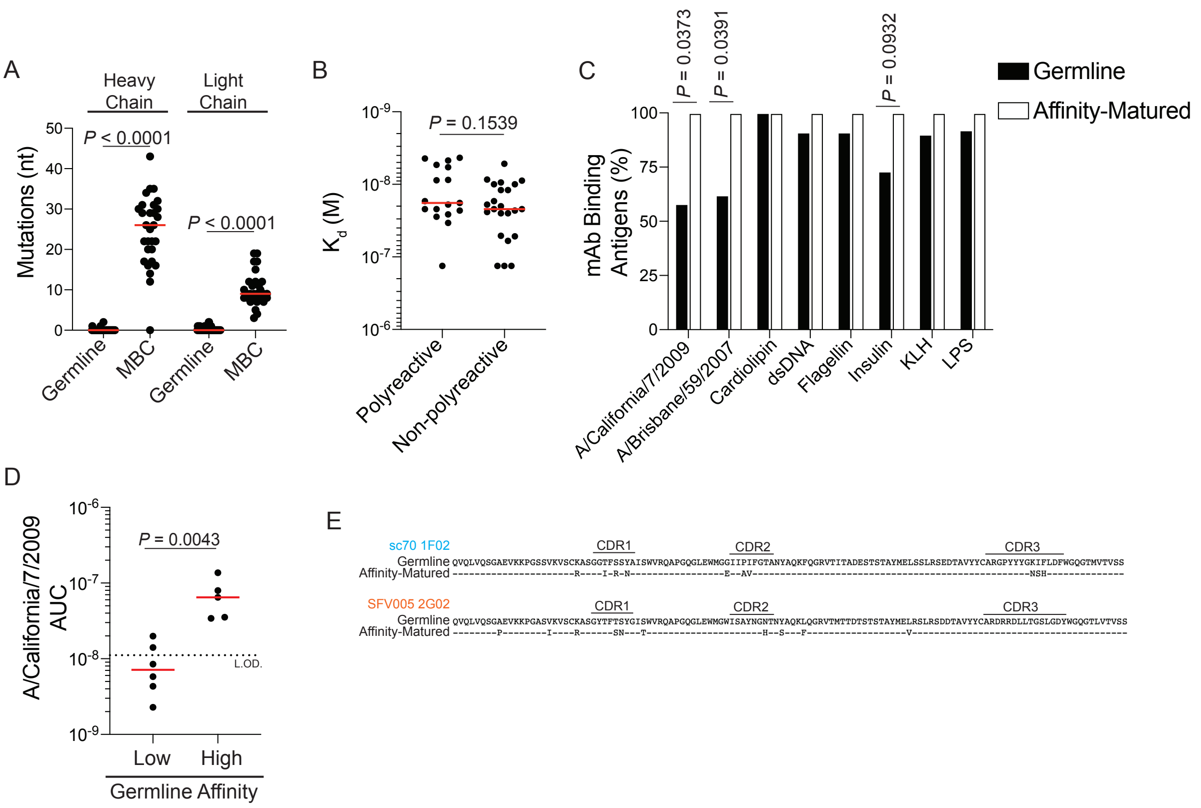

Figure S6: Germline precursors of broadly-reactive antibodies are polyreactive. Related to Figure 6. (**A**) Somatic hypermutations (nucleotide mutations) of heavy and light chains of stalk domain-binding germline (n=50) and MBC (n=29) mAbs tested in **Figure 6A**. (**B**) Affinity of polyreactive (n=17) and non-polyreactive (n=23) germline mAbs binding the stalk domain. (**C**) Proportion of reverted germline mAbs generated from affinity-matured polyreactive and corresponding affinity-matured mAbs binding influenza viruses and polyreactive panel antigens. (**D**) Area under the curve (AUC) of reverted germline mAbs categorized as high (n=5) or low (n=6) affinity binding to A/California/7/2009 related to **Figure 6E**. (**E**) Heavy chain sequences of germline and affinity-matured sc70 1F02 and SFV005 2G02. For data in **A**, **B**, and **D**, each symbol represents one mAb and the red bar indicates the median. Data in **A**, **B**, and **D** were analyzed by an unpaired non-parametric Mann-Whitney Test and data in **C** were analyzed by Fisher's Exact Test.

Table S1: Influenza vaccination and infection and influenza-negative naïve B cell and MBC cohorts. Related to STAR Methods.

Cohort	# of Subjects	# of mAbs	Average # mAbs per subject (range)	Reference
pH1N1 MIV	11	133	12 (1 – 29)	(Andrews et al., 2015a)
2010-2011 TIV	12	48	4 (1 – 6)	(Andrews et al., 2015a)
2014-2015 QIV	8	166	21 (8 – 53)	(Neu et al., 2019)
H7N9 LAIV/IIV	5	31	6 (2 – 16)	(Henry Dunand et al., 2016)
Elderly pre-H1N1 TIV	13	77	6 (1 – 21)	(Henry et al., 2019)
Chimeric HA Vaccine Germline	12	50	4 (1 – 16)	(Bernstein et al., 2019)
Chimeric HA Vaccine MBCs	12	29	2 (1 – 11)	(Bernstein et al., 2019)
2014-2015 H3N2 Infected	3	18	6 (2 – 10)	(Chen et al., 2018)
2015-2016 H1N1 Infected	4	21	5 (1 – 10)	(Chen et al., 2018)
Influenza-Negative Naïve B cells	3	52	17 (11 – 28)	(Duty et al., 2009)
Influenza-Negative MBCs	4	56	14 (7 – 22)	(Koelsch et al., 2007)

Table S2: Subject demographics for cohorts. Related to STAR Methods. *No demographic information was obtained from the H7N9 LAIV/IIV cohort and the influenza-negative naïve B cell and MBC cohorts.

Cohort	# of Subjects	Male (%)	Mean Age [Range]
pH1N1 MIV	11	36.4	41.1 (24 – 64)
2010-2011 TIV	12	58.3	29.3 (23 – 43)
2014-2015 QIV	8	37.5	29.9 (24 – 34)
Elderly pre-H1N1 TIV	13	53.8	75.7 (71 – 89)
Chimeric HA Vaccine Germline	12	25	27.7 (20 – 37)
Chimeric HA Vaccine MBCs	12	25	30.9 (24 – 36)
2014-2015 H3N2 Infected	3	66.7	43 (34 – 49)
2015-2016 H1N1 Infected	4	31.25	31.3 (23 – 46)
